# Food marketing, eating and health outcomes in children and adults: a systematic review and meta-analysis

**DOI:** 10.1017/S0007114524000102

**Published:** 2025-03-28

**Authors:** Emma Boyland, Magdalena Muc, Anna Coates, Louisa Ells, Jason C. G. Halford, Zoe Hill, Michelle Maden, Jamie Matu, Maria J. Maynard, Jayne Rodgers, Victoria Targett, Mimi Tatlow-Golden, Michelle Young, Andrew Jones

**Affiliations:** 1 Department of Psychology, University of Liverpool, Liverpool, L69 7ZA, UK; 2 Faculty of Wellbeing, Education and Language Studies, The Open University, Milton Keynes, MK7 6AA, UK; 3 Obesity Institute, Leeds Beckett University, Leeds, LS1 3HE, UK; 4 School of Psychology, University of Leeds, Leeds, LS2 9JT, UK; 5 Office for Health Improvement & Disparities, Department of Health and Social Care, London, SW1H 0EU, UK; 6 Liverpool Reviews and Implementation Group, University of Liverpool, Liverpool, L69 3GB, UK; 7 Teesside University International Business School, Teesside University, Middlesbrough, TS1 3BX, UK

**Keywords:** Food marketing, Eating, Health, Meta-analysis, Children, Adults, Systematic review

## Abstract

The marketing of unhealthy foods has been implicated in poor diet and rising levels of obesity. Rapid developments in the digital food marketing ecosystem and associated research mean that contemporary review of the evidence is warranted. This preregistered (CRD420212337091)^1^ systematic review and meta-analysis aimed to provide an updated synthesis of the evidence for behavioural and health impacts of food marketing on both children and adults, using the 4Ps framework (Promotion, Product, Price, Place). Ten databases were searched from 2014 to 2021 for primary data articles of quantitative or mixed design, reporting on one or more outcome of interest following food marketing exposure compared with a relevant control. Reviews, abstracts, letters/editorials and qualitative studies were excluded. Eighty-two studies were included in the narrative review and twenty-three in the meta-analyses. Study quality (RoB2/Newcastle–Ottawa scale) was mixed. Studies examined ‘promotion’ (*n* 55), ‘product’ (*n* 17), ‘price’ (*n* 15) and ‘place’ (*n* 2) (some > 1 category). There is evidence of impacts of food marketing in multiple media and settings on outcomes, including increased purchase intention, purchase requests, purchase, preference, choice, and consumption in children and adults. Meta-analysis demonstrated a significant impact of food marketing on increased choice of unhealthy foods (OR = 2·45 (95 % CI 1·41, 4·27), Z = 3·18, *P* = 0·002, I^2^ = 93·1 %) and increased food consumption (standardised mean difference = 0·311 (95 % CI 0·185, 0·437), Z = 4·83, *P* < 0·001, I^2^ = 53·0 %). Evidence gaps were identified for the impact of brand-only and outdoor streetscape food marketing, and for data on the extent to which food marketing may contribute to health inequalities which, if available, would support UK and international public health policy development.

Poor diet is recognised as a major risk factor for ill health and premature death^(1,2)^. In 2018, just 28 % of adults and 18 % of children (5–15 years) in England were eating the recommended five portions of fruits and vegetables a day, and mean intake of free sugars and SFA exceeded recommendations in all age groups^(3)^. Further, few people were meeting targets for salt, fibre and excess energy intake^(4,5)^. Between 2006 and 2020, voluntary government salt, sugar and energy reduction programmes were introduced in the UK as part of a comprehensive strategy to encourage reformulation of products contributing substantially to intakes of the targeted nutrients and where reformulation is possible^(5–8)^.

About two-thirds (64 %) of adults in England are living with overweight or obesity^(9)^, and one in three children leave primary school (aged 11 years) with overweight or obesity^(10)^. As of 2020, the annual full cost of obesity in the UK has been estimated at £58 billion (about 3 % of the UK’s Gross Domestic Product), with an estimated £6·5 billion spent each year on treating obesity-related disease^(11)^. Obesogenic food environments, characterised in part by the presence of extensive marketing of foods and beverages (hereafter: foods) high in saturated fat, sugar and/or salt (HFSS), are thought to be a key driver of rising levels of obesity worldwide^(12)^. The recent National Food Strategy acknowledged that most marketing money is spent promoting unhealthy (HFSS) products, with 32 % of spend on brand-only advertising^(13)^. Digital advertising spend in the UK, as globally, is substantial and growing^(14)^.

The WHO has set out recommendations and frameworks to guide Member States in the development and implementation of policies to restrict food marketing^(15–17)^. While implementation of the WHO recommendations has been inconsistent^(18,19)^, a recent systematic review and analysis of implemented policies demonstrated that mandatory policies (more often than voluntary measures from industry) can achieve meaningful reductions in food marketing exposure and power, as well as reducing purchasing of unhealthy foods^(20)^.

Chile’s 2016 Food Labelling and Advertising law banned all unhealthy food marketing ‘directed to’ or ‘intended for’ children under 14 years, including via the Internet. A second phase extended the television advertising restrictions to cover 06.00–22.00 on all channels. Evaluations indicate this has effectively reduced children’s exposure to unhealthy food advertising^(21)^ and may have contributed to declines in unhealthy food consumption in young children^(22)^. In 2020, the UK Government launched a new obesity strategy^(23)^ which included a number of measures designed to ‘help people live healthier lives’, including energy labelling for out-of-home food businesses, consultations on front-of-pack and alcohol energy labelling, and HFSS volume promotion and placement restrictions. Alongside these measures, the government announced its intention to ban HFSS products being marketed on TV before 21.00 and ban all paid-for HFSS food marketing online. The UK approach to legislation has been assessed to be comprehensive and more likely than other approaches (such as those in Chile and Canada) to meet its regulatory objectives^(24)^, but implementation has been delayed until October 2025.

Over recent decades, increasing evidence has been accrued to demonstrate that the marketing and advertising practices of transnational food companies affect the attitudes, preferences, choices and eating behaviours of children^(25–28)^ and adolescents^(29)^, as well as shaping social and cultural norms for entire populations^(30)^. Evidence supports ‘a hierarchy of effects’ of food marketing, that is, that exposure sequentially affects immediate outcomes such as attitudes and behaviours, and later weight-related outcomes^(31)^.

However, much of this evidence relates to television food advertising^(31)^ or individual forms of digital media such as advergames^(32)^ or social media^(33)^. Television food advertising, while still extensive worldwide^(34)^, may no longer be the dominant form of marketing exposure for young people as time spent using digital media has overtaken that of TV viewing^(35)^. In recent years, there has been extensive digital media take up and use in adults and children in the UK^(35,36)^. Previous reviews have noted that understanding of the impact of food marketing in all digital spaces is crucial^(8,37)^, particularly when sociodemographic differences in digital media use suggest the potential for it to widen health inequalities^(38,39)^. A 2015 evidence review by Public Health England (PHE)^(8)^ reported on the findings of forty-five primary research articles on food marketing published between 2010 and 2014; here, it was noted that there was a dearth of evidence of impact of marketing from new and emerging strategies such as digital media and sponsorship. Furthermore, many reviews focus only on the effects on young people^(28)^, and not adults, which has implications for the comprehensiveness of health impact assessments for food marketing policies^(40)^.

The aim of this systematic review was to provide an updated synthesis of the evidence since 2014 for behavioural and health impacts of food marketing on both children and adults, using the 4P’s framework (Promotion (e.g. advertising), Product (e.g. product design and packaging), Price (e.g. price-setting and discounts) and Place (e.g. location of products)). The 4Ps are frequently identified by systems mapping activities as being critical to successful obesity prevention at the population level^(41)^. The framework was used for consistency with, and to build directly on, the previous PHE review that adopted this approach^(8)^.

## Methods

This systematic review was preregistered on the PROSPERO database (CRD420212337093) and was reported in line with Preferred Reporting Items for Systematic Reviews and Meta-Analyses (PRISMA)^(42)^ and MOOSE^(43)^ guidelines (see online Supplementary File 1 for completed checklists) and the systematic review quality criteria of the updated AMSTAR tool^(44)^. An amendment to the original protocol is described in online Supplementary File 2. The methods used in the current review are largely consistent with those used in the 2015 review^(8)^, although there are some differences in the search terms and inclusion criteria between the two reviews, reflective of the differing scopes (e.g. the focus solely on sugary foods and drinks in the earlier review, and inclusion of outcomes relating to dental health and non-communicable disease).

### Search strategy

Searches were undertaken in MEDLINE, CINAHL, APA PsycInfo, Web of Science (all databases), EMBASE, ERIC, The Cochrane Library (Cochrane Database of Systematic Reviews, Cochrane Central Register of Controlled Trials), Health Management Information Consortium, Communication & Mass Media Complete and Academic Search Complete using a comprehensive search strategy where search terms were refined from^(8)^ to reflect the updated scope (online Supplementary File 2). Searches sought to identify studies added to databases from 1 October 2014 to January 2021 (i.e. recent evidence published after that included in ref. 8). Searches were conducted by an experienced information specialist (M.M. (Maden)). We conducted focused searches across grey literature, including key government and organisation websites, and Google Scholar. We also hand-searched the reference lists of relevant reviews captured by the searches and eligible articles. For publications with insufficient or missing information, email contact was attempted with corresponding authors a maximum of two times, three weeks apart.

### Study selection

To be considered for inclusion, studies were required to meet the eligibility criteria set out in the Participant, Intervention/exposure, Comparator, Outcome, Study Design (PICOS) framework (Table 1). Studies were included if they provided quantitative data on one or more of the outcomes of interest (as defined in Table 1) in humans (any age) following exposure to unhealthy (as defined by study authors, relevant descriptors included HFSS, ultra-processed, discretionary and ‘high-in’ products) food marketing compared with a relevant comparator exposure. Types of articles included were primary data articles reporting experimental studies of quantitative or mixed-method design (including randomised controlled trials (RCT), pre-post designs and quasi experimental studies), observational studies (cross-sectional or longitudinal) and modelling/simulation studies. Excluded study and article types were qualitative studies, reviews, conference abstracts, dissertations, editorials, and letters to the editor.

**Table 1.** PICOS: inclusion and exclusion criteria of the review

Component	Included	Excluded
Population	Human populations of any age	Animal studies Studies published before 2014 and/or included in ref. 8
Intervention/exposure	Food or non-alcoholic beverage marketing, as defined by WHO^*^	Marketing for food supplements, vitamins or infant formula Studies solely assessing effects of non-marketing interventions (e.g. television viewing in general, self-efficacy programmes), or non-commercial campaigns (such as public health education initiatives).
Comparison	No marketing, non-food or beverage marketing, less food or beverage marketing or less powerful (fewer techniques) food or beverage marketing	
Outcome	Outcomes of interest measured at any relevant time (e.g. pre- and post-marketing exposure): Choice or intended choice of food or non-alcoholic beverage (defined as the selection of items over others) Consumption (defined as energy, sugar, total food and/or nutrient intake or nutritional quality of diet) Purchasing/sales or intended purchasing of food or non-alcoholic beverage products (defined as the buying of items, intentions to buy items or sales data) Preferences (defined as greater liking of items over others, inclusive of brand preference, taste preference and product preference) Product requests (‘pester power’) or intended requests (defined as requests that an item be purchased, or intentions to request that an item be purchased) Body weight/BMI/obesity (defined as the amount a person weighs, weight status or a measure to indicate weight status)	Studies not reporting on any of the outcomes of interest
Study design/publication type	Primary studies or reports Experimental studies of quantitative or mixed-method design Observational studies Modelling/data simulation studies	Purely qualitative studies Reviews of studies Dissertations/theses Abstracts (including conference) Editorials Letters
Language/origin	English language Data from OECD country	

OECD, Organisation for Economic Co-operation and Development.

* WHO (2010). Set of recommendations for the marketing of food and non-alcoholic beverages to children. http://whqlibdoc.who.int/publications/2010/9789241500210_eng.pdf

Articles retrieved from searches were uploaded into Endnote (Clarivate, Philadelphia, 2013) and duplicates removed. The Endnote library was exported to Covidence (Veritas Health Innovation) for screening in two phases: (i) title/abstract screening and (ii) full text screening. Screening of each record was undertaken independently by at least two researchers from a pool of five (M.M. (Muc), J.M., J.S., A.C. and E.B.). Disagreements on inclusion or exclusion of studies were resolved through discussion, if necessary, a third reviewer was consulted. The reasons for exclusion of studies at full text were recorded.

### Data extraction

Data extraction was undertaken by one researcher and independently checked in full by a second (M.M. (Muc), J.S., A.C. and E.B.) using standardised data extraction templates. Disagreements were resolved through discussion. The following data were extracted: reference (author, year and country), study funding, conflicts of interest, study design and methods, participant characteristics (where possible data relating to equity characteristics according to PROGRESS-Plus^(45)^), relevant outcome measures and effect estimates (e.g. OR and risk ratios).

### Quality assessment

The quality of included studies was assessed by one reviewer and independently checked in full for agreement by a second. Quality assessment was undertaken using the Cochrane risk of bias (RoB2) tool for RCT^(46)^ and Newcastle–Ottawa scale for non-randomised studies^(47)^. Modelling/simulation studies did not undergo a quality assessment due to the lack of an appropriate tool given the heterogeneity of study design and focus.

### Data analysis

The decision was taken to not conduct meta-analyses where there were too few available effect sizes (≤ 5) to create a robust pooled effect size or to explore sources of heterogeneity. Analyses with small numbers of effect sizes would be more adversely affected by outlying effects which might lead to incorrect conclusions being drawn. We conducted meta-analytic power analysis for the effect on consumption using the ‘power.analysis’ function from the ‘dmetar’ package. We assumed a pooled effect of standardised mean difference (SMD) about 0·37, with twenty effect sizes, and approximately twenty in each condition, and the presence of heterogeneity, based on previous work^(25)^. This would provide us 95·6 % power.

Random effects restricted maximum likelihood estimator analyses were conducted for consumption and choice outcomes using the ‘metafor’ package in R^(48)^. We fit multilevel meta-analytic models to account for some studies providing more than one effect size. The I^2^ statistic was used to assess heterogeneity of study results, with a value of I^2^ = 50 % or more indicating important or substantial heterogeneity. Where necessary, OR were converted to SMD using the formulas set out by Polanin and Snilstviet^(49)^. These were handled using the R Package ‘effectsize’ and the specific command ‘odds_to_d’. Where data were not available in text but were presented in a figure, we used WebPlotDigitizer^(50)^ to extract the relevant information. We also converted SMD to a common language effect size to aid interpretation^(51)^. Further details (and a link to the data and analysis scripts) are provided in online Supplementary File 3.

## Results

### Study selection

**Fig. 1.**
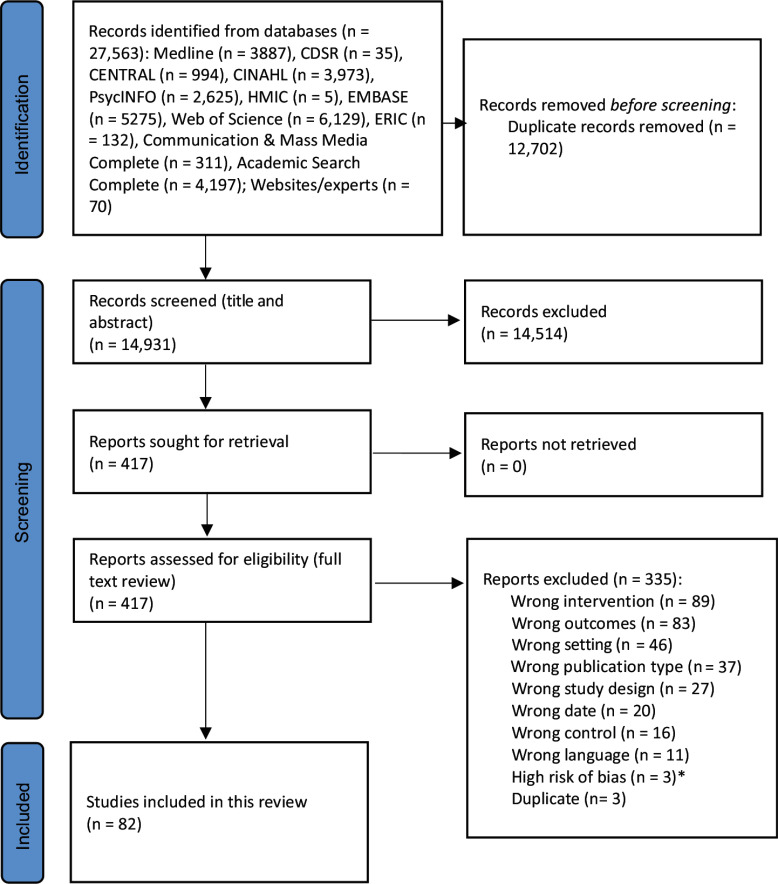
PRISMA flow chart. PRISMA, Preferred Reporting Items for Systematic Reviews and Meta-Analyses. *Papers authored by Professor Brian Wansink have been excluded on the grounds that they have a high risk of bias. To date, fifteen of his studies have been retracted because of academic misconduct^1^, and at least one of the studies retrieved by the searches has been found to have substantial flaws^2^. ^1^ https://www.vox.com/science-and-health/2018/9/19/17879102/brian-wansink-cornell-food-brand-lab-retractions-jama. ^2^ https://www.lockhaven.edu/∼dsimanek/pseudo/cartoon_eyes.htm.

The search identified 14 931 unique titles after the removal of 12 702 duplicates. In total, 417 articles were eligible for full text screening. From this, eighty-two were included in the narrative review, of which twenty-three were also in the meta-analyses (Fig. 1).

### Study characteristics

Details of all included studies, including outcomes of quality assessment, are provided in Table 2. Most (*n* 55) examined the food marketing in the context of ‘promotion’, *n* 17 explored ‘product’, *n* 15 studied ‘price’ and *n* 2 reported on ‘place’-based marketing (not mutually exclusive, some studies feature in more than one category). Not including modelling studies, publications reported on studies with child (*n* 48), adult (*n* 19), and child and adult participants (*n* 9, of which *n* 3 reported on retail sales data at the household level or above). The studies measured the relationship between food marketing and food consumption (*n* 38), choice (*n* 20), preferences (*n* 13), purchasing (*n* 12), purchase requests (*n* 3) and body weight (*n* 8) (some studies reported on more than one relevant outcome, all were included).

**Table 2.** Characteristics of included studies

Author (date), country	RoB/QA rating	Study design and population	Marketing format and exposure	Outcome(s) measured	Key finding(s) relating to the outcome(s) of interest for this review
Promotion					
Agante (2019), Portugal	3	NRS Experimental, *n* 104 children 6–9 years	Digital, advergame by leading potato crisp brand or no advergame	Preference Choice	Exposure to an advergame based on a leading potato crisp brand resulted in significantly greater brand preference (*P* < 0·001), product preference (*P* = 0·035), brand choice (*P* < 0·001) and product choice (*P* = 0·046) relative to the control group who had completed a questionnaire with no advertising exposure.
Anderson (2015), USA	Some concerns	RCT, *n* 50 children 9–14 years	Television advertising, food or non-food	Consumption	No significant differences in consumption in boys (*P* = 0·2) or girls (*P* = 0·28).
Arendt (2015), Austria	Some concerns	RCT, *n* 134 children 7–11 years	Television advertising, food or non-food	Choice	Significantly greater proportion of children in the group exposed to the food advertising chose the advertised chocolate product compared with the control group (37 % *v*. 21 %, *P* = 0·04).
Baldwin (2018), Australia	6	NRS Observational, *n* 417 children 10–16 years	Digital, watching food and beverage brand videos on YouTube or not	Consumption	Children who self-reported watching food and beverage brand videos on YouTube had a higher unhealthy food and drink combined score *v*. non-watchers (B = 0·80, *P* = 0·003).
Boyland (2015), England	Some concerns	RCT, *n* 59 children 7–10 years	Television advertising, food or non-food	Preference Choice	Children exposed to fast-food ad greater preference for fast food *v*. those who had seen toy ads (4·2 ± 1·1 *v*. 3·8 ± 1·2, *P* = 0·004). No significant differences in brand preference (*P* > 0·99) or kilocalorie load of items chosen from hypothetical menu (*P* = 0·961).
Boyland (2017), England	5	NRS Experimental, *n* 55 adults 20–62 years	Television advertising, food or non-food	Consumption	Exposure to food commercials did not affect food intake overall (*P* > 0·05) or in the lean (*P* > 0·05) or overweight subgroups (*P* > 0·05) *v*. non-food commercials.
Boyland (2018), UK	5	NRS Observational, *n* 2471 children 7–11 years	Television and online advertising, high (> 3 h/d) or low (< 0·5 h/d) commercial viewing hours or Internet use	Purchase requests Purchasing Consumption Body weight	Those self-reporting high (> 3 h/d) *v*. low commercial TV viewing hours (< 0·5 h/d) showed greater odds of pestering for advertised foods (OR 2·82, *P* < 0·05), purchasing advertised foods (OR 2·93, *P* < 0·001), consuming sugary drinks (OR 2·63, *P* < 0·001) and living with overweight/obesity (OR 1·59, *P* = 0·002). Those self-reporting spending more (> 3 h/d) *v*. less (< 0·5 h/d) time on the Internet had greater odds of pestering for advertised foods more often (OR 2·51, *P* < 0·001), purchasing advertised foods (OR 3·81, *P* < 0·001) and living with overweight/obesity (OR 1·79, *P* < 0·001).
Bragg (2019), USA	Low risk	RCT, *n* 1503 adolescents 14 years	Television advertising, racial targeting congruent or incongruent with participant race	Purchase intention	No significant effect on reported likelihood of purchasing the advertised product (*P* = 0·67).
Brown (2017), USA	6	NRS Experimental, *n* 114 children 9–11 years	Product placement, movie with high or low product placement	Choice Consumption	Children who saw high product placement movie had 3.1 times the odds of choosing featured snack *v*. those who saw low product placement movie (OR 3·07 (95 % CI 1·31, 7·18), *P* = 0·01). No difference in consumption (769·9 kcal ± 23·7 *v*. 804·2 kcal ± 23·8, *P* = 0·8).
Bruce (2016), USA	5	NRS Experimental, *n* 23 children 8–14 years	Television advertising, food or non-food	Preference	Exposure to food commercials enhanced taste preference ratings for test foods (four-point Likert) *v*. non-food commercials (0·68 *v*. 0·63, *P* < 0·05).
Buchanan (2017), Australia	Some concerns	RCT, *n* 60 young adults 20 years	Digital, brand websites and social media sites for two popular energy drink brands or relatively healthy nut bar brands	Purchase intention Consumption intention	Participants in experimental group showed greater purchase (*P* < 0·05) and consumption intention (*P* = 0·005) towards energy drinks *v*. control group.
Buchanan (2018), Australia	5	NRS Observational, *n* 359 adults 18–24 years	Digital, exposure and engagement and other non-digital formats (broadcast, print, in-store, sponsorship and endorsement)	Consumption	More frequent engagement with digital marketing (OR 1·47 (95 % CI 1·02, 2·10), *P* = 0·04) but not more frequent exposure alone (*P* = 0·78) associated with greater odds of being an energy drink user (*v*. less frequent engagement or exposure). More frequent exposure to other non-digital marketing formats not associated with greater odds of being an energy drink user (*P* = 0·58).
Coates (2019a), England	Low risk	RCT, *n* 176 children 9–11 years	Digital, mock Instagram influencer profiles with food or non-food images	Consumption	Children who viewed social media influencers with unhealthy foods consumed more unhealthy snacks (384·83 kcal ± 141·21 *v*. 292·24 ± 146·85, *P* = 0·001) and food overall (448·3 ± 140·82 *v*. 357·1 ± 146·5, *P* = 0·001) *v*. those who viewed influencers with non-food products.
Coates (2019b), England	Some concerns	RCT, *n* 151 children 9–11 years	Digital, YouTube influencer videos with unhealthy or healthy foods or non-food products	Consumption	Children who viewed social media influencers with unhealthy foods consumed significantly more snacks than children who viewed the influencers with non-food products, but only when ad disclosure message was present (309·03 ± 105·65 *v*. 260·3 ± 71·86, *P* = 0·03).
Critchlow (2020), UK	5	NRS Observational, *n* 3348 children and adolescents 11–19 years	Multiple formats, newspapers, magazines, television, catch-up/streaming services, billboards, radio and social media	Consumption	Consumption of sugary drinks greater for those with higher self-reported unhealthy food marketing exposure *v*. low or medium exposure (AOR = 2·3, *P* < 0·001).
Dalton (2017), USA	7	NRS Observational, *n* 548 children 4 years	Television advertising, child targeted fast-food ads	Consumption	Consumption of fast food in previous 7 d was associated with exposure to child-targeted fast-food TV ads (RR 1·26 (95 % CI 1·13, 1·41), *P* < 0·05).
Dixon (2018), Australia	Some concerns	RCT, *n* 1132 young adults 18–24 years	Sports sponsorship, promotional video with sponsor content for unhealthy food brand or non-food brand	Choice	The proportion of participants choosing the sponsor brand product over the non-sponsor product was not significantly different between conditions (*P* = 0·0531).
Dixon (2020), Australia	Some concerns	RCT, *n* 1613 adults	Television advertising, conventional or pseudo-healthy confectionery advertising or non-food	Choice	Exposure to conventional and pseudo-healthy confectionery advertising led to greater choice of advertised brand *v*. non-food advertising (29·4 % *v*. 9 %, *P* < 0·01 and 27 % *v*. 11·8 %, *P* < 0·01).
Domoff (2021), USA	5	NRS Observational, *n* 190 adolescents 14 years	Television advertising, commercial viewing time	Body weight	Commercial TV viewing time not significantly associated with body weight (BMI percentile) overall or in sex subgroups (all *P* > 0·05).
Dovey (2017), England	Low risk	RCT, *n* 80 young adults 20 years	Television advertising, food or non-food	Choice	Exposure to commercials for HFSS food *v*. non-food did not affect choice of HFSS snack packs in participants with high or low dietary restraint (*P* > 0·05).
Egbert (2020), USA	7	NRS Experimental, *n* 38 young adults 18–19 years	Television advertising, food or non-food	Consumption	Exposure to unhealthy television food advertising did not affect candy consumption *v*. non-food advertising overall (*P* = 0·108) or in those with high dietary restraint (*P* = 0·181). Binge-eaters consumed more candy than non-binge-eaters after food advertising (28·29 g ± 14·21 *v*. 17·94 g ± 11·96, *P* = 0·022), no such difference after non-food advertising (*P* = 0·111).
Emond (2016), USA	Some concerns	RCT, *n* 60 children 4 years	Television advertising, food or non-food	Consumption	Exposure to unhealthy snack ads led to greater energy intake (126·8 kcal *v*. 97·3 kcal, *P* = 0·04) *v*. exposure to department store ads.
Emond (2019a), USA	6	NRS Observational, *n* 624 children 4 years	Television advertising, fast-food ads	Consumption	No significant association between children’s exposure to advertising by a leading fast-food brand and their risk of consumption of that brand across the whole sample (RR = 1·14 (95 % CI 0·95, 1·36), *P* > 0·05) or in children whose parents consumed fast food more frequently, but advertisement exposure increased the risk of consumption 2-fold in children whose parents consumed fast food less frequently (RR = 1·97 (95 % CI 1·20, 3·22), *P* < 0·01).
Emond (2019b), USA	7	NRS Observational, *n* 624 children 4 years	Television advertising, high-sugar breakfast cereal ads	Consumption	Children with either recent (within prior 7 d) or recent and past exposure to high-sugar breakfast cereal TV advertisements had an increased risk of brand-specific high-sugar breakfast cereal intake (RR = 1·34 (95 % CI 1·04, 1·72), *P* < 0·05 and RR = 1·37 (95 % CI 1·15, 1·63), *P* < 0·001, respectively).
Folkvord (2017), Spain, Netherlands	Some concerns	RCT, *n* 211 children 6–12 years	Digital, advergame promoting energy dense snacks or non-food products	Consumption	Children exposed to advergame promoting energy-dense snacks consumed more energy *v*. children exposed to advergame promoting non-food products in the Netherlands sample overall (182·43 ± 137 *v*. 90·27 ± 129·1, *P* < 0·001, medium-large effect d = 0·69) and in both the younger (6–8 years, medium-large effect d = 0·79, *P* = 0·01) and the older subgroups (9–11 years, medium-large effect d = 0·66, *P* = 0·03). In the Spain sample, no such effect overall (*P* = 0·417) or in the younger subgroup (*P* > 0·05) but there was in the older subgroup (medium effect d = 0·51, *P* = 0·01).
Forde (2019), UK, Canada, Australia, USA, Mexico	8	NRS Observational, *n* 15 515 adults	Digital SSB promotion, traditional (TV, radio, text, magazine and newspaper) and combined formats	Consumption	Exposure to digital sugar-sweetened beverage (SSB) promotion associated with increased likelihood of high SSB consumption *v*. non-consumption (RRR = 1·52 (95 % CI 1·34, 1·71)). Exposure to traditional SSB promotion associated with increased likelihood of high SSB consumption *v*. non-consumption (RRR = 1·40 (95 % CI 1·26, 1·56)). Similar effect found for self-reported food marketing exposure via traditional, digital, recreational and functional formats combined (RRR = 1·13 (95 % CI 1·11, 1·16)).
Gatou (2016), Greece	8	NRS Experimental, *n* 183 children 11 years	Television advertising, food or non-food	Preference	No difference in children’s food preferences after food advertising *v*. non-food advertising (*P* = 0·37).
Giese (2015), Finland and Germany	5	NRS Observational, *n* 2851 adults and children 8–21 years	Television advertising	Consumption Body weight	Unhealthy food advertising exposure positively associated with weekly fast-food consumption overall (B = 0·90, *β* = 0·18, *P* < 0·001) and in the Finnish and German subsamples (*P* < 0·001). No significant associations with body weight (*P* > 0·05).
Gilbert-Diamond (2017), USA	Some concerns	RCT, *n* 172 children 9–10 years	Television advertising, food or non-food	Consumption	Children exposed to food advertisements consumed more of advertised snack *v*. those exposed to the toy advertisements (*P* < 0·01), but no difference in the consumption of the non-advertised snack foods or total snack intake between conditions (*P* = 0·21 and *P* = 0·98).
Gregori (2017), Italy	Some concerns	RCT, *n* 16 children 6–11 years	Television advertising, food or non-food	Consumption	No significant difference in post-viewing snack intake (*P* > 0·05).
Ham (2016), USA	4	NRS Experimental, *n* 322 adults	Digital, advergame for unhealthy or healthy food brands	Purchase intention	Purchase intention significantly lower in those exposed to a commercial unhealthy food brand advergame *v*. those exposed to a commercial healthy food brand advergame (2·724 ± 1·375 *v*. 3·677 ± 1·58, *P* < 0·001).
Hennessy (2015), USA	5	NRS Observational, *n* 347 parents of children 3–16 years	Multiple formats, SSB advertising on TV, radio, print, billboards and Internet	Consumption	Parents’ weekly exposure to SSB advertising positively associated with their soda consumption (*P* < 0·05) but not their children’s (*P* > 0·05).
Heredia (2017), Portugal	2	NRS Observational, *n* 60 children 8–12 years	Television advertising and viewing time	Consumption Body weight	Greater television viewing positively associated with higher frequency of fast-food consumption for school days (r_s_ = 0·54, *P* < 0·001) and weekends/holidays (r_s_ = 0·51, *P* < 0·001). Similar associations found between television viewing times and body weight, for school days (r_s_ = 0·51, *P* < 0·001) and weekends/holidays (r_s_ = 0·55, *P* < 0·001).
Kearney (2020), England	Some concerns	RCT, *n* 101 children 8–10 years	Television advertising, food or non-food	Consumption	Children consumed more energy after food ads compared with non-food ads (566·4 kcal ± 229·9 *v*. 518·0 kcal ± 255·7, *P* = 0·007), difference in responding between weight status subgroups was significant, *P* = 0·037). No interaction between intake response and socio-economic status (*P* > 0·05).
Kelly (2015), Australia	5	NRS Observational, *n* 417 children 10–16 years	Television advertising, commercial viewing time	Consumption	Positive association between amount of commercial television viewing and mean total unhealthy food and drink score (*P* = 0·001). Evidence of a dose–response relationship: link between television viewing and poor diet was strongest for those who watched the most commercial television.
Kumar (2017), USA	N/A	Television advertising Social media In-store promotions	Impact of TV and social media advertising and in-store promotions	Sales	Social media has positive and significant static effect on brand sales (*B* = 0·088, *P* < 0·01) four times greater than that of television advertising (*B* = 0·021, *P* < 0·10). In-store promotion deals estimated to have a greater impact on brand sales than television advertising (*B* = 0·878, *P* < 0·01).
Lazard (2018), USA	Some concerns	RCT, *n* 301 adults (study 1) and *n* 200 adults (study 2)	Static advertising images, either enhanced photo manipulation advertisement for food or basic non-enhanced photo of food	Preference Purchase intention	Product preferences were greater in those who viewed the enhanced advertisement image *v*. basic image in both studies (4·94 ± 1·01 *v*. 4·4 ± 1·24, *P* < 0·001 and 5·1 ± 1·2 *v*. 4·35 ± 1·23, *P* < 0·01, respectively). Purchase intentions only significantly greater in study 2 (4·26 ± 1·17 *v*. 3·83 ± 1·33, *P* < 0·05).
Lorenzoni (2017), Chile	Some concerns	RCT, *n* 8 children 6–12 years	Television advertising, food or non-food	Consumption	No significant difference in subsequent snack consumption (*P* = 0·89).
Masterson (2019), USA	Some concerns	RCT, *n* 41 children 7–9 years	Television advertising, food or non-food	Consumption	No significant effect on subsequent food consumption (*P* = 0·4).
Matthes (2015), Austria	Some concerns	RCT, *n* 121 children 6–14 years	Product placement movie with high or low snack product placement	Consumption Preference	Children in high product placement group were more likely than those in no product placement group to consume advertised snack *v*. two similar snacks provided (44·7 % *v*. 18·4 %, *P* < 0·05). No difference between the groups in preferences towards the brand (*P* > 0·05) or product (*P* > 0·05).
Naderer (2018a), Austria	7	NRS Experimental, *n* 363 children 6–15 years	Product placement, movie with no or static or interactive product placement	Choice	Relative to those who saw no product placement, children who saw both static (51·2 % *v*. 13·5 %, *P* < 0·001) and interactive placement (62·6 % *v*. 13·5 %, *P* < 0·001) more likely to choose target brand over alternatives.
Naderer (2018b), Austria	Some concerns	RCT, *n* 130 children 6–11 years	Product placement, movie with static image snack product placement or placement integrated into plot	Choice Preference	Children who viewed integrated product placement were more likely to choose the target brand over alternatives (29·5 % *v*. 16·3 %, *P* < 0·001) but did not show greater preference towards the brand (*P* > 0·05) *v*. the control group.
Neyens (2017), Belgium	Some concerns	RCT, *n* 940 children 6–14 years	Television advertising, food or non-food Digital advergame or no advergame	Product request intent Preference	No difference in pester intent between the two TV advertising groups (*P* = 0·363). Greater proportion of children who played advergame showed preference for advertised brand *v*. control group (75 % *v*. 67 %). No difference in pester intent between the two advergame groups (*P* = 0·363).
Newman (2020), UK	5	NRS Observational, *n* 3394 children and adolescents 11–19 years	Multiple formats	Consumption	High HFSS marketing exposure *v*. low associated with greater chip/fried potato consumption (OR = 2·18 (95 % CI 1·76, 2·70), *P* < 0·001).
Norman (2018), Australia	Low risk	RCT, *n* 154 children 7–12 years	Television and digital (advergames), food or non-food advertising	Consumption	No significant differences for TV advertising (all *P* > 0·05). For combined TV and advergame group, energy intake greater on food advertising days *v*. non-food advertising days both at snack (2168 kJ ± 787 *v*. 1968 ± 698 (95 % CI 80, 308), d = 0·3, *P* = 0·001) and at snack and later lunch meal combined (*P* = 0·001). Same effect found for both weight status subgroups (*P* < 0·05).
Nguyen (2017), USA	7	NRS Observational, state level health data	Digital, energetic density of food tweets	Body weight	Significant relationship found between the energetic density of food tweets and state level obesity (B = 1·78 (95 % CI 0·89, 2·67), *P* < 0·01).
Ponce-Blandon (2020), Spain	Some concerns	RCT, *n* 421 children 4 years	Movie, with or without unhealthy food advertisements embedded	Choice	No effect of food advertisement exposure on choice of the advertised product *v*. alternative (*P* = 0·8803).
Powell (2017), USA	7	NRS Observational, *n* 8340 children 10–11 years and 13–14 years	Television advertising, soft drink, SSB and cereal advertising	Consumption Body weight	Exposure to sugary beverage advertisements positively associated with higher frequency of consumption (coefficient 0·294 ± se 0·094, *P* = 0·001). Significant and positive association between cereal advertising exposure and BMI percentile (coefficient 0·410 ± se 0·164, *P* < 0·05). Significant and positive association between cereal advertising exposure and percent body fatness (coefficient 2·368 ± se 0·571, *P* = 0·001).
Putnam (2018), USA	Some concerns	RCT, *n* 132 children 4–5 years	Digital, bowling game with character with or without unhealthy snack	Choice	Number of healthy or unhealthy snacks chosen was not different between groups (*P* = 0·35).
Redondo (2020), Chile	Some concerns	RCT, *n* 812 children and adults < 18–41 years +	Product placement, movie with or without fast-food brand scene and dialogue	Choice	Those who viewed movie with the product placement scene were more likely to choose the advertised *v*. alternative brand than those who viewed the movie without that scene (*P* < 0·001).
Royne (2017), USA	4	NRS Experimental, *n* 64 children 6–11 years	Product placement, television cartoon with or without product placement for a cola beverage	Choice	More children from the group exposed to product placement selected cola (*n* 11 (73·3 %) *v*. *n* 9 (60 %).
Smit (2020), Belgium	3	NRS Observational, *n* 453 children 8–12 years	Digital, frequency of watching online video blogs	Consumption	Self-reported frequency of watching vlogs was related to consumption of unhealthy beverages 2 years later (b = 0·15, s e = 0·07, 95 % CI 0·02, 0·28, *P* = 0·021). The analyses did not yield significant relations for beverages over a 1-year period, nor for snacks consumption over a 1- or 2-year period (all *P* > 0·05).
Smith (2020), Australia	Some concerns	RCT, *n* 156 children 7–12 years	Digital, web-based game with rewarded video advertising for an unfamiliar confectionery brand or no advertising	Choice Consumption	Greater proportion of children exposed to food advertising chose promoted snack *v*. those in control group (64·1 % *v*. 19·5 %, *P* < 0·002). Condition did not influence overall energy intake measured in grams (*P* = 0·78) or kilocalories (*P* = 0·46).
Tarabashkina (2016), Australia	Low risk	RCT, *n* 354 children 7–13 years	Digital, pop-up webpage advertisements for cookies or non-food	Choice	No difference between conditions in proportion of children who selected the advertised biscuit (*P* = 0·63).
Velazquez (2016), Canada	4	NRS Observational, *n* 82 children 8–15 years	Multiple formats, including billboards, inside/outside stores, magazines, television	Choice Preference	Greater *v*. lower exposure to food and beverage advertising associated with significantly increased choice of (B = 0·62, *P* = 0·004) but not preference for (*P* = 0·18) unhealthy foods.
Yau (2021), England	6	NRS Observational, *n* 1552 adults	Digital advertising and digital food delivery service advertising Multiple formats (traditional, digital, recreational and functional)	Body weight	Exposure to digital and digital food delivery service advertising associated with increased odds of obesity (OR = 1·80 (95 % CI 1·33, 2·44), *P* < 0·001 and OR = 1·40 (95 % CI 1·05, 1·88), *P* < 0·01 respectively). Those with self-reported unhealthy food advertising exposure across traditional only or traditional, digital, recreational, or functional environments did not have greater odds of obesity than those with no exposure.
Product					
Aerts (2019), Belgium	8 (Study 1) 5 (Study 2)	NRS Experimental, *n* 47 children 6 years (study 1), *n* 24 children 5 years (study 2)	Front-of-pack portion size, large or small	Consumption	Exposure to large *v*. small portion size image resulted in greater snack consumption (67·5g ± 36·81 *v*. 60·04g ± 33·2, *P* = 0·013) but no difference in consumption of the unhealthy snack between conditions (*P* = 0·986) (study 1). Exposure to large *v*. small portion size image resulted in greater consumption of chocolate spread on the first slice offered (9·94g ± 6·38 *v*. 7·63g ± 5·2, *P* = 0·009) but not total consumption (*P* = 0·398) (study 2).
Bialkova (2016), Netherlands	Some concerns	RCT, *n* 240 adults	Front-of-pack claims, taste benefit or none	Purchase intention	Purchase intention higher for products with no claim *v*. front-of-pack taste benefit claims (*P* < 0·001).
Bollard (2016), New Zealand	6	NRS Experimental, *n* 604 children and adults 13–24 years	Brand imagery on pack or plain pack	Preference	Plain packaging (*v*. branded packaging) had negative impact on preference for SSB (*P* < 0·001).
Dixon (2017), Australia	Some concerns	RCT, *n* 904 children 5–9 years	Toy premiums, unhealthy meals paired with or not paired with the toy	Choice Purchase requests	When unhealthy meals paired with toy premium, 80 % of children selected unhealthy meal *v*. 71 % when not paired with a toy premium (*P* = 0·001). No difference for likelihood of the child requesting the meal from parents (*P* = 0·370).
Girju (2019), USA	6	NRS Observational, *n* 24 800 adults	Pack size, family or single serve	Consumption	Potato crisp consumption greater from family size *v*. single-serve packs (*B* = 0·491, *P* < 0·001).
Gregori (2017), Italy	Some concerns	RCT, *n* 16 children 6–11 years	Brand imagery on test food packaging, present or absent	Consumption	No difference in snack consumption found between conditions (*P* > 0·05).
Heard (2016), USA	Some concerns	RCT, *n* 51 children 7–12 years	On-pack promotions, competition based or none	Purchasing (hypothetical)	Competition-based *v*. no promotions on unhealthy items did not affect quantity ‘purchased’ from online simulated grocery store (*P* = 0·86).
Leonard (2019), USA	5	NRS Experimental, *n* 26 children 6 years (study 1), *n* 139 children 7 years (study 2) and *n* 110 children 5 years (study 3)	On-pack licensed characters	Choice Consumption	Children more likely to choose snack with licensed character present *v*. no character (77 % *v.* 23 %, *P* = 0·006 and 73 % *v.* 27 %, *P* = 0·033, respectively). No effect on consumption (*P* = 0·97).
Mann (2018), USA	4	NRS Experimental, *n* 76 children 11–14 years	Store version of packaging or school ‘copycat’ equivalent	Purchase intention	Purchase intention greater for store version of a snack packaging *v*. school ‘copycat’ equivalent (6·62 ± 2·68 *v*. 5·86 ± 3·02, *P* < 0·05).
McDarby (2016), Ireland	Some concerns	RCT, *n* 404 children 8–13 years	Personalised SSB drink bottles	Choice	Odds of choosing unhealthy drink greater when drinks had child’s name on label *v*. no label control (OR = 2·34 (95 % CI 1·16, 5·55), *P* = 0·024).
McGale (2016), England	Some concerns	RCT, *n* 60 children 7 years (study 1), *n* 149 children 6 years (study 2)	Front-of-pack brand equity characters	Preference	The presence of brand equity characters on product packaging (*v*. with no character) increased taste preference (both *P* < 0·001) and snack choice within product pairs (*P* < 0·001 for congruent associations and *P* = 0·001 for incongruent) but not final snack choice (from all six products, *P* = 0·06 and *P* = 0·935, respectively).
Neyens (2015), Belgium	Some concerns	RCT, *n* 22 children 4 years	Front-of-pack portion size, large or small	Consumption	Children exposed to large *v*. small image consumed more cereal (20·59g ± 4·99 *v*. 15·93g ± 5·31, *P* < 0·0001).
Ogle (2017)	Some concerns	RCT, *n* 149 children 6–9 years	On-pack promotional character	Choice	Greater choice of test items in control condition (character absent) *v*. intervention condition (character present; 63·5 % *v.* 29·8 %).
Reimann (2017), USA	5	NRS Experimental, *n* 109 children 8 years	Toy premium, present or absent	Choice	Presence of toy led to greater choice of the test item *v*. no toy (*P* < 0·001).
Schumacher (2020), Netherlands	7	NRS Experimental, *n* 58 young adults 18–19 years	On-pack labels, ‘surprise’ or regular	Choice	Chances of participant choosing larger serving size greater when snacks labelled with ‘surprise’ *v*. regular label (*P* < 0·001).
Talati (2018), Australia	Some concerns	RCT, *n* 1953 adults and children 10–65 years	On-pack claims, health or none	Choice	Health claim *v*. no claim did not affect probability of choosing the product (0·19 (95 % CI 0·17, 0·20), *P* > 0·05).
Werle (2016), France	Some concerns	RCT, *n* 166 adults 20 years	Original chocolate packaging or plain packaging	Purchase intention Consumption	Purchase intention lower for plain packaging (2·52 ± 1·95 *v*. 4·46 ± 1·89, *P* = 0·001). No difference in consumption between original (130·96g ± 115·98) and plain pack conditions (111·98g ± 93·34, *P* = 0·499).
Price					
Cohen (2015), USA	4	Audit of 40 food outlets, self-report survey of 980 adults	SSB price reductions	Body weight	No association between exposure to SSB price reductions and BMI (B = 0·029, *P* > 0·05).
Guan (2019), USA	3	NRS Observational, *n* 2500 households	Discount coupons	Purchasing	Households with coupons for convenience foods had greater purchase rate *v*. households without coupons (1·32 *v*. 0·15, *P* < 0·001).
Harris (2017), USA	Some concerns	RCT, *n* 191 adults	Price promotion, hypothetical restaurant menus	Purchase intention	Participants exposed to menu with price promotion had greater purchase intention (*B* = 1·37, *P* = 0·05) and (hypothetical) consumption (*B* = 273·88, *P* < 0·01) *v*. those exposed to menu without price promotion, although direct effect on purchase intention no longer significant (*P* = 0·11) after consumption norms were accounted for.
Mamiya (2018), Canada	N/A	Price discounting	Impact of store-level price discounting	Sales	Across all three area-level education levels and type of store (supermarkets, pharmacies, supercenters and convenience stores), discounting positively associated with soda sales. Discounting in pharmacies was associated with greater increases in purchasing in areas with the lowest educational attainment compared with areas with higher education, similar effect seen for convenience stores but to a lesser extent.
Mathe-Soulek (2016), USA	7	NRS Observational (sales data)	Price promotions, fast-food outlets	Sales	Number of price-based promotions not related to change in same-store sales (*P* > 0·05) when economic and seasonal conditions effects were controlled for, but there was a small significant correlation between the number of items on ‘new product’ price promotions and same-store sales (*P* < 0·05).
Place					
Cohen (2015), USA	4	Audit of 40 food outlets, self-report survey of 980 adults	Low-nutrient food displays	Body weight	Exposure to low-nutrient food displays (SSB and foods high in solid oils, fats and added sugars) had positive association with BMI (*B* = 0·002, *P* < 0·05).
Ejlerskov (2018), England	9	NRS Observational (sales data)	Marketing at supermarket checkouts, comparison of stores with and without restrictive policies	Sales	Supermarkets that did not have policies to restrict the marketing of less-healthy foods at checkouts sold more less-healthy food packages at 4 weeks (157 700 packages (95 % CI 72 700, 242 800)) and 12-months (185 100 packages (95 % CI 121 700, 248 500)) post-policy implementation *v*. supermarkets with restrictive policies in place.
Brown (2018), Australia	N/A	Television advertising	Legislation to restrict HFSS advertising before 21.30	Consumption Body weight	The intervention would reduce children’s energy intake by an average of 115 kJ/d (approximately 27·5 kcal) and BMI by an average of 0·352 kg/m^2^. Benefits would be greater in most disadvantaged children (–132 kJ/d and −0·395 kg/m^2^) than least disadvantaged (–97 kJ/d and −0·299 kg/m^2^).
Dubois (2018), UK	N/A	All formats	Total ban on advertising for crisps in UK market	Sales Purchasing	Pre-ban, total monthly expenditure on crisps was £100·85m (95 % CI 99·78, 101·91) and the total quantity sold per month was 14·8m Kg (95 % CI 14·64, 14·98 mKg). With prices held constant in the model, the estimated impact of the ban would be a 15·1 % reduction in expenditure to £85·62m (95 % CI 82·44, 88·26) and a 15·2 % fall in quantity sold to 12·55m Kg (95 % CI 12·05, 12·97). Reductions in purchasing anticipated to have an impact on health, with the ban estimated to lead to 15·2 % reduction in the total quantity of energy purchased by households, from 313·70bn kJ (95 % CI 310·22, 316·37) to 265·94bn kJ (95 % CI 256·46, 274·18).
Lopez (2015), USA	N/A	Television advertising	Direct and ‘spillover’ effects of television advertising on brand-level consumer demand for carbonated soft drinks (CSD) in the USA. The impact of TV advertising restrictions.	Purchasing Sales	A 1 % increase in advertising spend on Coke regular increases demand for Coke regular by 0·7982 % and reduces demand for Diet Coke by 0·0193 %. Analysis including spillover effects shows that a 1 % increase in advertising spend for Diet Coke increases demand for Coke regular by 0·2901 % as well as increasing demand for Diet Coke by 0·3971 %. All advertising for CSD being prohibited would lead to a decline in the market share of all CSD (e.g. Coke regular from 2·36 % to 1·81 %) and a concurrent increase in market share for alternatives (e.g. fruit juice, bottled water and milk) from 86·72 % to 89·54 %.
Mytton (2020), UK	N/A	Television advertising	Legislation to restrict HFSS advertising before 21.00	Consumption Body weight	If all HFSS advertising before 21.00 was withdrawn, the intervention would decrease daily energetic intake by 9·1kcal (95 % UI 0·5–17·7kcal) which would reduce the number of UK children (5–17 years) with overweight (including obesity) by 3·6 % (95 % UI 1·1–7·4 %). The estimated reduction in obesity was approximately 2-fold greater among children in the least affluent social grade compared with the most affluent.

RoB, risk of bias; QA, quality assessment.

QA scores ≤ 4 = unsatisfactory quality, 5–6 = satisfactory quality, 7–8 = good quality, 9–10 = very good quality.

RoB, risk of bias; QA, quality assessment; NRS, non-randomised studies; RCT, randomised controlled trial; HFSS, high in fat, sugar and/or salt; RR, risk ratio; RRR, relative risk ratio; AOR, adjusted odds ratio.

Publications reported on RCT (*n* 37), experimental non-randomised studies (NRS, defined as experimental studies where authors did not explicitly describe random allocation of participants to conditions; *n* 15), observational NRS (*n* 24, of which seven were longitudinal and the rest were cross-sectional) and modelling/simulation studies (*n* 6) ranging in size from small to very large (*n* 17 to 24 800 participants). Four simulation studies^(52–55)^ provided insight into the impact of food marketing by examining the potential effect of restrictive policies, and these are described separately (online Supplementary File 4).

Included studies provided evidence from Australia (*n* 11), Austria (*n* 4), Belgium (*n* 3), Canada (*n* 2), Chile (*n* 2), Finland and Germany (*n* 1), France (*n* 1), Greece (*n* 1), Ireland (*n* 1), Italy (*n* 1), Netherlands (*n* 3), Netherlands and Spain (*n* 1), New Zealand (*n* 1), Portugal (*n* 2), Spain (*n* 1), the UK (England *n* 8, UK-wide *n* 7), and the USA (*n* 32). Notably, all of these are high-income countries, and no included studies were conducted in low-income or middle-income economies.

Funding sources were declared for 55/82 publications. Of these, one study^(56)^ was funded by the American Academy of Advertising, but no other explicit commercial funding was declared. All other funding was derived from research councils, banks, universities, charities, foundation trusts or government. Nine of the fifty-five publications declared that no specific funding had been received for the research.

Risk of bias assessments indicated that almost all RCT (*n* 32) had ‘some concerns’ of bias, with the remaining five RCT deemed to have low risk (online Supplementary File 5 Table S1). This largely reflected a lack of specific information in publications on the randomization procedure used, allocation concealment and any deviations from the intended interventions. Of the NRS (online Supplementary File 5 Tables S2–3), nine were deemed to be of unsatisfactory quality, twenty-one were satisfactory quality, eleven were good quality and one was very good quality. Quality issues in NRS related mostly to limited information being provided about participant sampling and non-respondents, while experimental studies tended to achieve higher scores due to the controlled exposures to marketing and objective measurement of outcomes compared with observational studies using self-report measures. As this review focused on a topic of contemporary public health policy relevance, all included study designs can be considered ‘good’ evidence through the lens of appropriateness^(57)^. However, it is notable that in both evidence hierarchies in evidence-based medicine^(58)^ and the GRADE approach to assessing the certainty of available evidence (as applied to food marketing and eating behaviour in ref. 28, RCT are assumed to be a ‘better’ standard of evidence than observational studies. As such, an RCT with some concerns of bias would (within such frameworks) typically be considered more robust or ‘certain’ evidence than a high-quality observational study.

The results are presented as a narrative summary for each of the 4Ps followed by the quantitative syntheses for food consumption and choice. Due to the number of studies on ‘promotion’ and ‘product’, these sections are further subcategorised by marketing format. Evidence is also organised by study type and age of participants (child, adolescent and adult) and where relevant, greater prominence is given to studies of better quality. Given the volume of studies included in this synthesis, effect sizes (where reported in articles) could not always be provided in the text but are all in Table 2. Terminology is used as reported by study authors, if required further details and definitions can be found by consulting individual papers.

### Promotion

#### Television advertising

Twenty-nine publications explored the impact of television food advertising, of which seventeen report an impact of food marketing on an outcome of interest for this review.

##### Studies with children: randomised controlled trials (*n* 12)

All studies had some concerns of bias except one^(59)^ which was low risk. Significant effects of television food advertising on relevant outcomes were reported in five of the RCT with children (participant ages ranged from 4 to 14 years), specifically that following exposure to television food advertising participants showed significantly greater preference for fast food^(60)^, greater choice of advertised products over alternatives^(61)^ and increased consumption of the advertised foods and/or general snacks^(62–64)^. Eight of the RCT with children reported that there was no statistically significant effect of television food advertising on intention to request products^(65)^, food brand preferences^(60)^, choice of advertised foods over alternatives^(66)^, or hypothetical^(60)^ or actual food consumption^(59,67–70)^.

##### Studies with children: experimental studies (*n* 2)

One satisfactory quality experimental study in children (8–14 years) found significant effects of television food advertising on food preference, specifically enhanced taste preference for the test foods^(71)^, while a good quality study reported no difference in 11-year-old children’s food preferences after food advertising exposure compared with after non-food advertising^(72)^.

##### Studies with children: observational studies (*n* 7)

Of the seven observational studies conducted with children (age range 4–16 years), three were good quality. Two of these reported that television food advertising exposure was significantly associated with increased consumption of fast food (cross-sectionally)^(73)^ and high-sugar breakfast cereals (longitudinally)^(74)^ in pre-school children. The third good quality observational study^(75)^ analysed data from a longitudinal survey of 10–14-year-olds and reported that exposure to soft drink and sugar-sweetened beverage (SSB) advertisements was significantly associated with higher frequency of soft drink consumption even when unexplained heterogeneity was controlled for. A significant association was also reported between food advertising exposure and greater body fatness and BMI percentile^(75)^.

Three observational studies were of satisfactory quality. Two of these studies reported significant associations between television food advertising exposure and greater odds of requesting purchase of (OR 2·82) or purchasing (OR 2·93) advertised foods^(76)^, greater consumption of unhealthy foods in general (OR 2·63)^(76)^, poorer diet quality (in a dose–response relationship)^(77)^, and greater odds of living with overweight or obesity (OR 1·59, *P* = 0·002)^(76)^. A 1-year longitudinal study reported a significant association between food advertising exposure and greater risk of fast-food consumption only in children whose parents consumed fast food less frequently (risk ratio (RR) = 1·97)^(78)^. One cross-sectional observational study was of unsatisfactory quality (see Table 2 for results)^(79)^.

##### Studies with children and adults: observational studies (*n* 1) and modelling studies (*n* 1)

A satisfactory quality observational study^(80)^ with children and adults (8–21 years) in Finland and Germany reported that unhealthy food advertising exposure was positively associated with weekly fast-food consumption, but there were no significant associations with body weight. A simulation identified that TV food advertising has a significant and strong effect in increasing demand for the advertised brand, and there is also a spillover effect whereby demand is also increased for other brands sold by the same company. Specifically, for direct effects the data showed that an increase in advertising spend on ‘regular’ Coke increases demand for that product and reduces demand for Diet Coke. The analysis including spillover effects showed that an increase in advertising spend for Diet Coke increases demand for both regular and diet Coke^(55)^.

##### Studies with adolescents: randomised controlled trials (*n* 1) and observational studies (*n* 1)

An RCT with low risk of bias reported no effect of exposure to TV food advertising in which the racial targeting was either congruent with participants (actors the same race as the participants) or not (actors a different race to the participants) on 14-year-old adolescents’ likelihood of purchasing the advertised product^(81)^, while a satisfactory quality cross-sectional NRS found no association between commercial TV viewing time and adolescent body weight (also 14 years)^(82)^.

##### Studies with adults: randomised controlled trials (*n* 2)

An RCT with low risk of bias reported that exposure to commercials for HFSS food products compared with non-food products did not significantly affect choice of HFSS snacks in participants with high or low dietary restraint^(83)^. An online RCT with some concerns of bias reported that exposure to both conventional (promoting sensory benefits) and pseudo-healthy (promoting sensory benefits and health attributes such as ‘made with real fruit’) confectionery advertising led to significantly greater choice of the advertised brand relative to non-food advertising exposure^(84)^.

##### Studies with adults: experimental studies (*n* 2)

Two studies (one good quality and one satisfactory) reported that exposure to television advertising for unhealthy foods did not affect subsequent food consumption in adults^(85,86)^.

#### Digital marketing: overall, websites, social media and influencers

Twelve publications explored the impact of digital food marketing, of which eleven report an impact of food marketing on an outcome of interest for this review.

##### Studies with children: randomised controlled trials (*n* 3)

Two RCT (one with low risk of bias and one with some concerns of bias) reported significant effects of social media influencer marketing, via Instagram and YouTube, respectively, on food consumption in 9–11-year-olds ^(87,88)^. The third RCT (low risk of bias) reported no significant difference in food choice, specifically selection of the advertised biscuit, between children (7–13 years) exposed to pop-up webpage advertisements for the biscuit or toys^(89)^.

##### Studies with children: Observational studies (*n* 3)

Two satisfactory observational studies in children (age range 7–16 years) reported significant associations between digital food marketing exposure and poorer diet quality^(90)^, greater odds of requesting (OR 2·51) or purchasing (OR 3·81) advertising foods^(76)^, and greater odds of living with overweight or obesity (OR = 1·79)^(76)^. The results of a longitudinal study of unsatisfactory quality^(91)^ are given in Table 2.

##### Studies with adults: randomised controlled trials (*n* 3)

All three RCT on digital food marketing with adult participants had some concerns of bias. One article described two RCT funded by the American Academy of Advertising, and it reported that digital food marketing exposure (an enhanced photo manipulation advertisement for food) increased product preferences and purchase intentions^(56)^. The other RCT reported that young adult participants exposed to the brand websites and social media sites of two popular energy drink brands showed greater purchase intention and intended consumption for energy drinks compared with the control group^(92)^.

##### Studies with adults: Observational studies (*n* 4)

A good quality observational international comparative study with adult participants from the UK, Canada, Australia, USA and Mexico^(93)^ found that increased self-reported exposure to digital SSB promotion was associated with an increased likelihood of high SSB consumption (relative risk ratio (RRR) = 1·52). A good quality observational social media analysis study reported a positive association between the energetic density of tweets (energy content per 100 g for all foods mentioned in posts on the digital platform Twitter) and obesity prevalence in the US state where the tweet originated^(94)^.

Across two satisfactory quality observational studies, it was reported that exposure to both digital food advertising and digital food delivery service advertising were associated with increased odds of obesity (OR = 1·80 and 1·40, respectively)^(95)^, but associations between digital marketing and greater odds of being an energy drink user (OR = 1·47) were only apparent for those with more frequent engagement (such as liking or sharing posts) and not more frequent exposure alone^(96)^.

#### Digital marketing: game-based

Six publications explored digital game-based marketing, of which four report an impact of food marketing on an outcome of interest for this review.

##### Studies with children: randomised controlled trials (*n* 4)

All RCT exploring digital game-based food marketing on outcomes in children (age range 4–14 years) had some concerns of bias. Two of these studies reported significant effects of digital game-based food marketing on preference for the advertised brand^(65)^ and choice of the advertised snack over alternatives but not increased energy intake^(97)^. A third RCT reported a significant effect of exposure to an unhealthy food advergame on energy consumption in a sample of children (6–12 years) from the Netherlands (medium-large effect d => 0·60 overall and in younger and older age subgroups separately) but only in the older subgroup (d = 0·51, medium effect) in the Spanish sample. Another RCT reported no effect of digital game-based food marketing on children’s choice of healthy or unhealthy items^(98)^.

##### Studies with children: experimental studies (*n* 1)

This experimental study with children (6–9 years)^(99)^ was deemed to be of unsatisfactory quality, and the results are given in Table 2.

##### Studies with adults: experimental studies (*n* 1)

This experimental study with young adults was also assessed as unsatisfactory quality^(100)^; see Table 2 for the results.

#### Product placement in movies

Six publications examined product placement in movies, all six report an impact of food marketing on an outcome of interest for this review. Three were RCT, all with some concerns of bias. Two RCT with children (ranging from 6 to 14 years) reported that exposure to food brand product placement significantly increased choice of the advertised snack^(101,102)^ but not attitudes towards the brand or product (i.e. whether they were ‘likeable’ and/or ‘funny’)^(101,102)^. Another RCT with both children and adults (< 18 years to 41 years and over) reported that those viewing a movie with product placement were significantly more likely to choose the advertised brand over an alternative brand than those who had seen the same movie with that scene removed^(103)^.

A good quality experimental study reported that product placement increased choice of the advertised snack over similar alternative snacks^(104)^, a satisfactory quality experimental study also reported effects on choice but not consumption^(105)^. A third experimental study was unsatisfactory quality^(106)^ (see Table 2 for results).

#### Sports-based marketing

One publication examined sports-based marketing; it did not report an impact of food marketing on an outcome of interest for this review. The RCT, with some concerns of bias, reported that exposure to the unhealthy food brand version of a promotional video for the 2018 Commonwealth Games (*v*. the non-food brand version) did not affect subsequent choice of the sponsored product in young adults^(107)^.

#### Multiple marketing formats

Nine publications examined the effects of multiple marketing formats together, including various combinations of TV, digital, radio, print (e.g. magazines), recreational (e.g. leisure environments) amd functional (e.g. school, work and retail environments) advertising formats. Seven report an impact of food marketing on an outcome of interest for this review.

One RCT (low risk of bias) reported that food consumption in children (7–12 years) was greater following a combination of television and online food advertising exposure (compared with non-food advertising exposure)^(59)^.

Seven observational studies (of which only one^(93)^ was good quality) reported on associations between combined advertising exposure from multiple sources and relevant outcomes. The good quality study reported greater exposure to food marketing to be associated with increased likelihood of high SSB consumption in an international sample of adults^(93)^. Two satisfactory quality observational studies reported greater combined food marketing exposure to be significantly associated with food consumption in 11–19-year-old adolescents^(108,109)^, whereas another reported significant associations with parents’ consumption only (not that of their children aged 3–16 years)^(110)^ or no significant relationship with consumption in adolescents (11–19 years)^(96)^ or odds of obesity^(95)^ in adults. The results of an observational study of unsatisfactory quality^(111)^ are given in Table 2.

Using a modelling approach, one study reported that social media and in-store promotions had significantly greater effects on brand sales than television advertising^(112)^.

### Product

#### Promotional characters

Three publications examined promotional characters on packaging, all three report an impact of food marketing on an outcome of interest for this review. Three RCT, all with some concerns of bias, explored the impact of promotional characters on food packaging on relevant outcomes in children (age range 4–9 years). Two reported that the presence (*v*. absence) of promotional characters on packaging increased taste preference^(113)^ and snack choice^(113)^. Conversely, another^(114)^ reported that there was greater choice of test items in the character absent condition relative to when the character was present. A single article described three satisfactory quality experimental studies where children (5–7 years) were reported to be significantly more likely to choose a snack when a licensed character was present (*v*. absent) on the packaging, but there were no effects on consumption^(115)^.

#### Product size

One publication examined product size; it reported an impact of food marketing on an outcome of interest for this review. The satisfactory quality observational study reported that adults’ crisp consumption was significantly greater from family size packs compared with single-serve packs^(116)^.

#### Other packaging characteristics (such as design, personalisation, labels, size and portion size imagery)

Eleven publications examined other packaging characteristics, of which nine report an impact of food marketing on an outcome of interest for this review.

##### Studies with children: randomised controlled trials (*n* 3)

All RCT had some concerns of bias and reported on outcomes in participants aged between 4 and 13 years. Choice of less healthy drinks was reported to be significantly greater when items had the child’s name added to the label (*v*. control; OR 2·34)^(117)^. The presence of competition-based promotions (*v*. no promotions) on the packaging of unhealthy items was not reported to affect the quantity of such items ‘purchased’ from an online simulated grocery store^(118)^. Children exposed to a large front-of-pack portion image were reported to consume significantly more cereal than those exposed to the small image^(119)^. There was no reported difference in snack intake between groups of children who had been exposed beforehand to the snacks in their own branded packaging or had seen them unbranded^(68)^.

##### Studies with children: experimental studies (*n* 2)

Across two experimental studies, children (5–6 years) exposed to packaging showing larger images of the food had significantly greater total snack consumption (good quality study) and significantly greater consumption of chocolate spread on the first slice but not overall (satisfactory quality study)^(120)^. The results of an unsatisfactory quality study with adolescents^(121)^ are in Table 2.

##### Studies with children and adults: randomised controlled trials (*n* 1)

In an RCT with some concerns of bias, probability of choice of the less healthy item was not significantly different whether a health claim was present or absent in either children or adults (10–65 years)^(122)^.

##### Studies with adolescents and adults: experimental studies (*n* 1)

In a satisfactory quality study, plain packaging (*v*. branded packaging) had a significant, negative impact on preference for SSB in teenagers and young adults (13–24 years)^(123)^.

##### Studies with adults: randomised controlled trials (*n* 2)

In two RCT with some concerns of bias, purchase intention was higher for products with no claim (*v*. a front-of-pack taste benefit claim)^(124)^ and lower for plain (*v*. original) packaging, but there was no significant difference in adults’ consumption^(125)^.

##### Studies with adults: experimental studies (*n* 1)

In a good quality experimental study, the chances of a young adult participant choosing a larger serving size was significantly greater when snacks were labelled with ‘surprise’ compared with a regular label^(126)^

#### Premium offers (toys provided with meals)

For studies of premium offers, one RCT with some concerns of bias reported that when unhealthy meals were paired with a toy premium, a significantly greater proportion of children (5–9 years) selected an unhealthy meal, compared with when unhealthy meals were not paired with a toy premium (80 % *v*. 71 %)^(127)^. However, there was no significant difference between conditions for likelihood of the child requesting the meal from parents. An experimental study of satisfactory quality^(128)^ reported that the inclusion of a toy with the smaller-sized meal, but not with the regular sized version, predicted smaller-sized meal choice in 9-year-old children.

### Price

Five publications examined ‘price’ marketing, of which four report an impact of food marketing on an outcome of interest for this review.

In an RCT with some concerns of bias, adults exposed to a menu with a price promotion had significantly greater purchase intention and (hypothetical) energetic consumption than those exposed to the menu without a price promotion^(129)^. A good quality observational study used objective data on price promotions and sales from fast-food chains and found that the number of price-based promotions was not significantly related to change in same-store sales when economic and seasonal conditions effects were controlled for, but there was a small significant correlation between the number of items on ‘new product’ price promotions and same-store sales^(130)^. Two further observational studies were of unsatisfactory quality ^(131,132)^; see Table 2 for results. A simulation study modelled weekly store-level sales of soda as a function of store-level price discounting, reporting that discounting in convenience stores was associated with greater increases in purchasing in areas with the lowest educational attainment (*v*. higher education levels), but effects were considered to be small (although test statistics were not reported)^(133)^.

### Place

Two publications examined ‘place’ marketing; both report an impact of food marketing on an outcome of interest for this review.

One very good quality observational study demonstrated that supermarkets that did not have policies to restrict the marketing of less-healthy foods at checkouts sold significantly more less-healthy food packages both 4 weeks (157 700 packages (95 % CI 72 700, 242 800)) and 12 months (185 100 packages (95 % CI 121 700, 248 500)) post-policy implementation compared with supermarkets with restrictive policies in place^(134)^. Another observational study was of unsatisfactory quality^(132)^, and results are shown in Table 2.

### Quantitative synthesis

Where possible, research evidence was quantitatively synthesized by outcome of interest (full details of methods and results including moderation analyses are given in online Supplementary File 3). Specifically, this type of synthesis was possible for the outcomes of consumption and choice, but not for purchasing/sales, preferences, product requests or body weight/BMI.

**Fig. 2.**
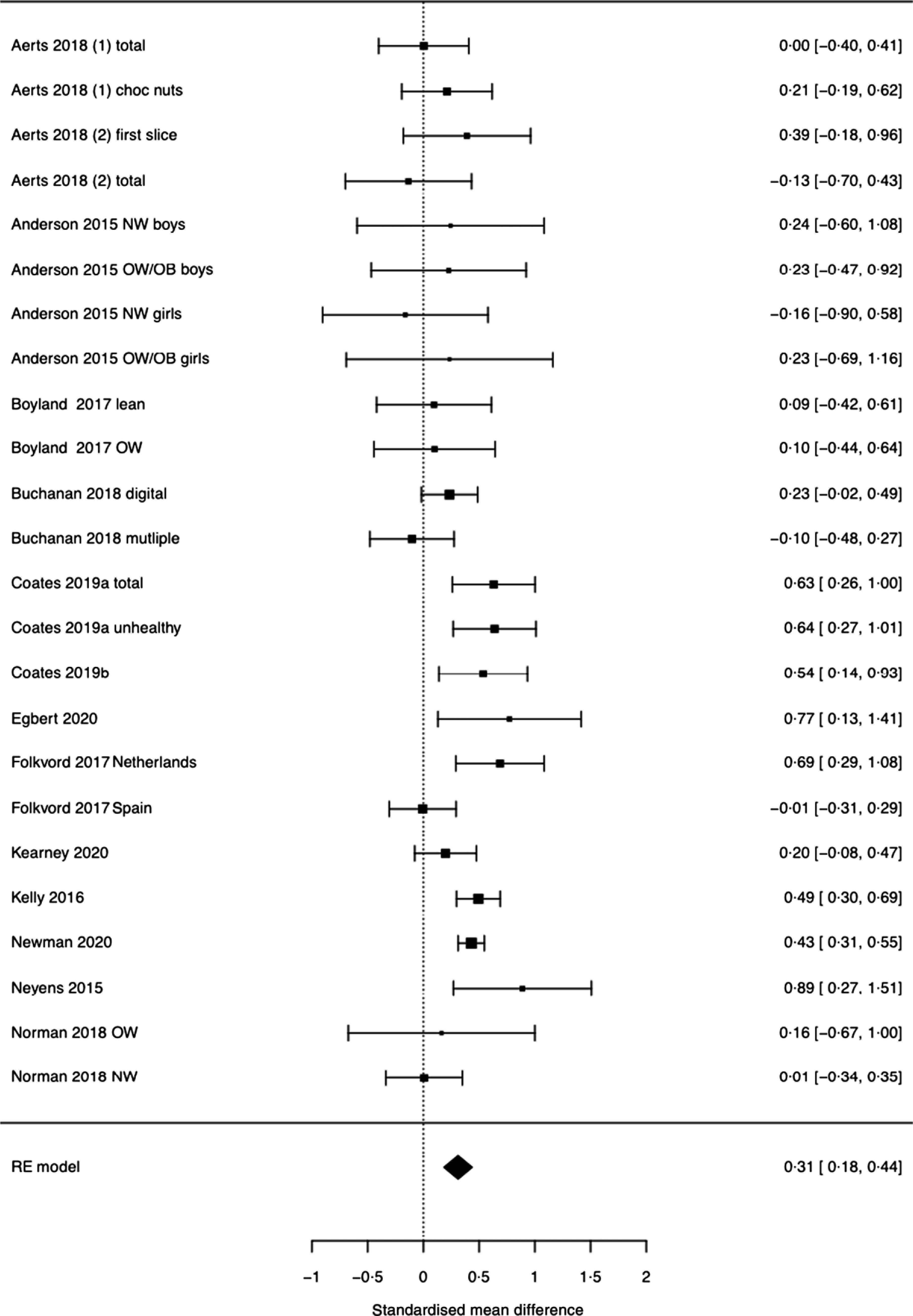
Forest plot for pooled analysis of the effect of food marketing on food consumption.

Food marketing exposure significantly increased food consumption (SMD = 0·311 (95 % CI 0·185 to 0·437), Z = 4·83, *P* < 0·001, I^2^ = 53·0 %) (Fig. 2). An SMD of 0·311 suggests that a person chosen randomly from an advertisement exposure group would be 58 % likely to consume more than a person chosen randomly from a control group. This also means that 62 % of individuals in the food advertisement groups will consume more than the control groups. The effect of food marketing on consumption was not significantly different across different marketing categories (4Ps), marketing formats (TV or digital), or by the age of participant sample (child or adult), or study quality. Additional analyses (P-curve) demonstrated that it was a likely to be a true effect (*P* < 0·001, online Supplementary Fig. S1).

**Fig. 3.**
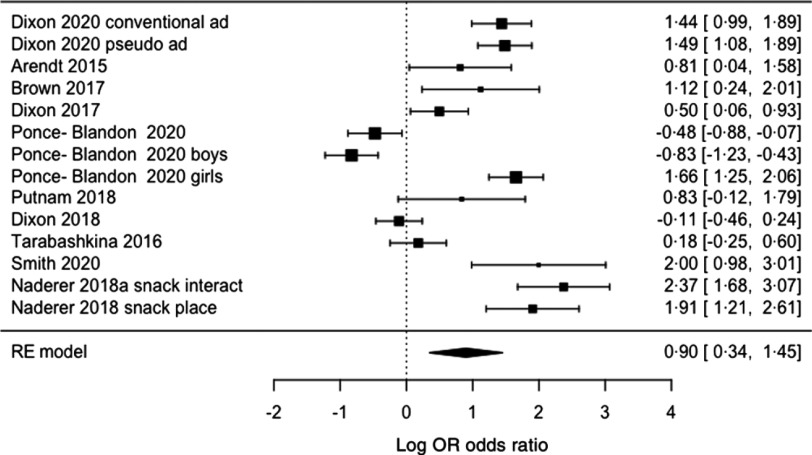
Forest plot for pooled analysis of the effect of food marketing on food choice. Explanatory note: studies assessed choice behaviour through participants pointing at images of foods, pointing at or picking up real food items, verbal choices, or hypothetical selection on paper or using computer-based tools.

Food marketing exposure also significantly increased choice of advertised items/unhealthy foods relative to alternative/control items (OR = 2·43 (95 % CI 1·40, 4·26), Z = 3·18, *P* = 0·002, I^2^ = 93·1 %) (Fig. 3). It was not possible to check for differences by marketing category, format, participant age or study quality, but P-curve analyses demonstrated that it was likely to be a true effect (*P* < 0·001, online Supplementary Fig. S2).

## Discussion

This review synthesized recent evidence from RCT and experimental and observational studies of impacts of food marketing on a range of eating and health outcomes in children and adults. It found that, while heterogeneous, there is evidence for food marketing impact on or associations with increased purchase intention, purchase requests, purchase, preference, choice, and consumption in both children and adults. While one study found a significant effect of food marketing on body fatness in children, data on body weight outcomes were relatively scarce.

The findings of this review are consistent with, and build upon, those of the previous PHE review on this topic in 2015^(8)^. It is not possible to direct compare effect sizes between the current review and the 2015 review, as the latter did not include meta-analyses. Here, meta-analytic models and P-curve analyses added to the body of evidence indicating the significant impact of food marketing on food choice and consumption in children and adults. These findings are consistent with those previously published for limited marketing forms (e.g. television advertising, advergames and social media) and outcomes (e.g. choice^(135)^ and intake ^(25,26,32,33)^) in child populations. The current work adds additional value through the inclusion of studies with adults, data studies focused on newer formats of digital marketing (e.g. gaming) and the P-curve analyses indicate that these are not a result of selective reporting or poor analytical practices but have evidential value.

The growth of literature on the impact of digital marketing in recent years is apparent. While in the 2015 review only seven studies were identified and all focused on advergames^(8)^, here seventeen digital marketing studies were identified, covering marketing impact from websites, social media, influencers and gaming. In both reviews, these studies demonstrated impacts of digital marketing exposures on outcomes such as food consumption, choice and preference. Although most studies included in the current review did not provide a direct comparison of the relative impact of digital marketing exposures compared with more traditional marketing approaches, previous analyses have suggested that the effect sizes for impact on diet-related behaviours are similar across both media^(28)^ and here there was also no moderating effect of marketing format for the consumption effect, that is, a subgroup analysis comparing the effect sizes for digital and TV marketing demonstrated no significant difference (results reported in the supplementary material).

Studies exploring product, price and place remain sparse (particularly relative to promotion), but the current review adds weight to what was previously known^(8)^. A total of twenty-one studies were identified for the 3Ps (minus promotion) in the previous review^(8)^; here, we add a further twenty-five recent studies published between 2014 and 2021. This additional evidence demonstrates that product and portion size impact on consumption, that promotional characters and other packaging characteristics affect preference and choice, and branding influences preferences and purchase intention. These overall findings are consistent with the previous review^(8)^ and other recent more focused reviews on the influence of packaging on consumer behaviour^(136)^. Furthermore, this review showed that both price promotion and place-based marketing impact food purchasing behaviours.

We were also able to draw upon the findings of more research conducted in the UK than was possible in the previous review (*n* 15 studies in this review *v*. *n* 5 previously^(8)^), which can support the development of UK policies based on the most relevant evidence for this population.

Several research gaps were identified. Most studies focused on school-aged children with a relative lack of data on impact of food marketing on pre-schoolers or older adolescents. There also remains a lack of evidence of impact of other food marketing approaches (such as outdoor sports-based marketing activities, and promotion via food delivery apps and video-on-demand services) and formats (e.g. audio advertising). There was also a lack of research evidence on the impact of brand-only marketing (where no product is shown, as distinct from product-based marketing). However, modelling data from Lopez *et al.* ^(55)^ provided useful insight by demonstrating that advertising for one brand appears to have a spillover effect on sales for other brands produced by the same company. This has implications for current UK policy proposals given that diet drinks can continue to be marketed under the new regulations. Few studies reported on impact findings disaggregated by sociodemographic characteristics (with gaps for sex including LGBTQAI+, weight status, socio-economic status and ethnicity), rather where these data were reported they were typically provided for descriptive purposes only or ‘adjusted for’ in analyses rather than group-specific analyses being undertaken. Therefore, even where these data were collected, it was not possible to demonstrate the extent to which food marketing may contribute to inequalities in health. This is also consistent with the previous review, where due to limited studies or heterogeneity of design, authors were unable to draw firm conclusions around differences by these characteristics. Future research should seek to address these limitations.

As with the previous review^(8)^, the evidence of food marketing impact is still dominated by studies on promotion, and this remains reliant on relatively small-scale experimental studies or RCT of typically moderate quality exploring acute effects and proximal outcomes (e.g. intake) rather than effects of repeated exposure (especially via multiple different media) or with outcomes such as body weight or health. It is important to acknowledge, however, that hierarchy of effects models of food marketing dispute the idea of there being simple direct links between marketing and these more distal outcomes and instead propose that food marketing operates both directly and indirectly with effects occurring in parallel and recurringly^(31)^. In addition, there is substantial complexity in the aetiology of obesity and notable methodological challenges in using health and metabolic measures beyond BMI or weight (e.g. nutritional deficiencies caused by inadequate diets) and in seeking to account for potential confounding factors in any study with body weight or related outcomes. Nevertheless, this is also a research gap that warrants attention. Similar hierarchical pathways have been proposed to explain the impact of alcohol marketing on consumption^(137)^ which, given the known energetic contribution of alcohol to weight gain and obesity^(138)^, may suggest that there is value in considering policies that act on food and alcohol together, or even more broadly^(139)^ to minimise migration of marketing from food to other harmful commodities.

This work has several strengths, including adhering to robust research integrity practices by using preregistration and the provision of open data and analysis scripts. However, the authors also acknowledge the following limitations. This review defined marketing as per the WHO framework for implementing the set of recommendations on the marketing of foods and non-alcoholic beverages to children^(16)^; therefore, evidence on the impact of other marketing (such as relating to distribution channels, business to business marketing, lobbying) and broader market strategies (for a review see^(140)^) was not included. It is also notable that there is considerable heterogeneity in the evidence base, likely to be a consequence of the large volume of studies and nuanced differences in study design (as has been noted elsewhere^(28)^) which can render it difficult to draw firm conclusions about the strength of evidence in some cases. Further, there are many studies using observational methods such as recall of marketing exposure that may be inaccurate^(141)^ and cross-sectional designs that do not facilitate inferences of causality. This review also did not explore moderation effects by country or region as this was beyond the aims of this research.

Findings from this review are consistent with the evidence from previous reviews that marketing has an impact on diet-related behaviours in both children and adults. There is now further evidence to support an effect for television food advertising on these outcomes, but also increasing data to demonstrate that exposure to digital food marketing has similar impacts on behaviours such as purchasing and consumption. There are trends towards greater investment in digital marketing approaches that are predicted to continue over the coming years in the UK, largely reflecting global patterns. Digital food marketing regulation is necessary, and the UK’s proposals establish a crucial principle while taking an important step towards reduced exposure to unhealthy food promotion online for children and adults in the UK. Research and policy attention towards brand marketing and new digital strategies is warranted, while robust studies on the impact of marketing via outdoor and sports-based marketing are also needed to inform public health policy development.

## Supporting information

Boyland et al. supplementary material 1 Boyland et al. supplementary material

Boyland et al. supplementary material 2 Boyland et al. supplementary material

Boyland et al. supplementary material 3 Boyland et al. supplementary material

Boyland et al. supplementary material 4 Boyland et al. supplementary material

Boyland et al. supplementary material 5 Boyland et al. supplementary material

## Supplementary material

For supplementary material/s referred to in this article, please visit https://doi.org/10.1017/S0007114524000102
