# Supplementary material for: Food marketing, eating and health outcomes in children and adults: a systematic review and meta-analysis
Source: Br J Nutr. 2025 Mar 28;133(6):781–805. doi: 10.1017/S0007114524000102 (PMC12169957; doi:10.1017/S0007114524000102)
Supplement: Boyland et al. supplementary material 2 — Boyland et al. supplementary material [file S0007114524000102sup002.docx]

**Example database search and grey literature sources**

We undertook an iterative approach to the development of the search strategy. Search terms were sourced through discussions with the review team, an exploratory search in PubMed Reminer (<https://hgserver2.amc.nl/cgi-bin/miner/miner2.cgi>) using search terms identified by the review team, scanning of search terms (free-text and associated thesaurus terms of database records) used in relevant topic systematic reviews and relevant terms included in known key relevant articles (including ^1^ and recent systematic review work by the research terms on the impact of food marketing on obesity). Search terms included relevant free-text, thesaurus and keywords and the use of advanced search operators (truncation and proximity operators).

We conducted sensitivity analyses to compare the retrieval of different search techniques to optimise the retrieval capability of the search strategy (e.g., search performance of proximity operators, phrase searching and field searching). We generated a set of relevant records to test the sensitivity of the search strategy. Searches were peer-reviewed (E.B., M.T.G., J.R.) for relevant terms and accuracy and agreed in consultation with OHID. In reporting the search strategies, we adhere to the PRISMA guidance (including the [PRISMA-S: PRISMA Search Reporting Extension](https://osf.io/ygn9w/)).

The following keywords form the main structure of the search: (food terms combined with OR) AND (advertising/marketing terms combined with OR) AND (health outcome/impact terms combined with OR). An example Medline search strategy is shown below. We translated search terms across multiple databases (accounting for uniqueness in thesaurus terms) and used targeted simplified free-text searches for grey literature sources.

Database: Ovid MEDLINE(R) ALL <1946 to January 08, 2021>

Search Strategy:

--------------------------------------------------------------------------------

1 exp Marketing/ (35368)

2 exp Advertising/ (15097)

3 exp Social Marketing/ (2409)

4 exp Advertising as Topic/ (15097)

5 advert*.ti,ab,kw. (18842)

6 market*.ti,ab,kw. (129498)

7 endors*.ti,ab,kw. (28175)

8 sponsor*.ti,ab,kw. (22778)

9 adspend*.ti,ab,kw. (2)

10 commercial.ti,ab,kw. (178986)

11 commercials.ti,ab,kw. (464)

12 brand*.ti,ab,kw. (21572)

13 (character or characters).ti,ab,kw. (106007)

14 in-game.ti,ab,kw. (758)

15 Influencer*.ti,ab,kw. (891)

16 twitter.ti,ab,kw. (3680)

17 "digital media".ti,ab,kw. (674)

18 instagram.ti,ab,kw. (689)

19 facebook.ti,ab,kw. (4074)

20 YouTube.ti,ab,kw. (2160)

21 Snapchat.ti,ab,kw. (105)

22 TikTok.ti,ab,kw. (17)

23 "online media".ti,ab,kw. (278)

24 exp Video Games/ (5727)

25 gaming.ti,ab,kw. (3606)

26 in-app.ti,ab,kw. (2782)

27 (mobile app or mobile apps).ti,ab,kw. (2879)

28 "spokes person".ti,ab,kw. (1)

29 spokesperson.ti,ab,kw. (169)

30 spokesm?n.ti,ab,kw. (175)

31 spokeswom?n.ti,ab,kw. (11)

32 "direct email*".ti,ab,kw. (35)

33 (celebrity or celebrities).ti,ab,kw. (1132)

34 cartoon*.ti,ab,kw. (1574)

35 stream*.ti,ab,kw. (71730)

36 smartphone*.ti,ab,kw. (13385)

37 exp Social Media/ (9033)

38 (Front-of-pack adj2 labels).ti,ab,kw. (86)

39 food label*.ti,ab,kw. (1483)

40 food packag*.ti,ab,kw. (2857)

41 ((food* or snack*) adj promotion).ti,ab,kw. (54)

42 "Product Placement".ti,ab,kw. (101)

43 Discount*.ti,ab,kw. (12772)

44 prize*.ti,ab,kw. (8179)

45 voucher*.ti,ab,kw. (2322)

46 BOGOF.ti,ab,kw. (0)

47 "buy one get one free".ti,ab,kw. (11)

48 or/1-47 (634517)

49 exp Food/ (1305719)

50 fast-food.ti,ab,kw. (3287)

51 junk-food.ti,ab,kw. (609)

52 takeaway*.ti,ab,kw. (428)

53 food.ti,ab,kw. (438584)

54 artificially sweetened beverages/ or exp carbonated beverages/ or energy

drinks/ or "fruit and vegetable juices"/ or sugar-sweetened beverages/ (5842)

55 exp Sugars/ (407669)

56 sugar*.ti,ab,kw. (127166)

57 sugar-sweetened.ti,ab,kw. (3142)

58 high-sugar.ti,ab,kw. (2163)

59 soft drink*.ti,ab,kw. (3914)

60 carbonated drink*.ti,ab,kw. (288)

61 energy drink*.ti,ab,kw. (1496)

62 fruit juice*.ti,ab,kw. (3986)

63 cordial.ti,ab,kw. (222)

64 fizzy.ti,ab,kw. (219)

65 sport* drink*.ti,ab,kw. (617)

66 confection?ry.ti,ab,kw. (952)

67 chocolate*.ti,ab,kw. (5458)

68 sweet*.ti,ab,kw. (32972)

69 jam.ti,ab,kw. (1751)

70 marmalade.ti,ab,kw. (72)

71 snack*.ti,ab,kw. (8309)

72 candy.ti,ab,kw. (1498)

73 soda*.ti,ab,kw. (5435)

74 cake*.ti,ab,kw. (5473)

75 biscuit*.ti,ab,kw. (1488)

76 dessert*.ti,ab,kw. (1493)

77 ice-cream*.ti,ab,kw. (1457)

78 pudding*.ti,ab,kw. (546)

79 yog?urt*.ti,ab,kw. (4699)

80 or/49-79 (1906057)

81 48 and 80 (79614)

82 ((food or fast-food* or junk-food* or snack* or sugar-sweetened or "soft drink*"

or "carbonated drink*" or "fizzy drink*" or "energy drink*" or "sport* drink*" or soda or

sweet* or takeaway or meal) adj promot*).ti,ab,kw. (169)

83 81 or 82 (79714)

84 exp Obesity/ (218590)

85 obese.ti,ab,kw. (130777)

86 obesity.ti,ab,kw. (260314)

87 overweight.ti,ab,kw. (72842)

88 "over weight".ti,ab,kw. (494)

89 (weight adj gain*).ti,ab,kw. (66612)

90 exp Adiposity/ or exp Body Weight/ (476358)

91 adiposity.ti,ab,kw. (25666)

92 BMI.ti,ab,kw. (151986)

93 "body mass index".ti,ab,kw. (192884)

94 exp Body Mass Index/ (130156)

95 exp Diet/ (286774)

96 nutrition.ti,ab,kw. (171042)

97 exp Energy Intake/ (46951)

98 exp Food Preferences/ (14706)

99 calorie*.ti,ab,kw. (26646)

100 calorific*.ti,ab,kw. (964)

101 ((food or snack* or sugar* or chocolate* or candy or sweet* or beverage* or

drink*) adj3 (consum* or eating or purchas* or request* or choice* or choose or

chose or prefer* or intake or pester*)).ti,ab,kw. (109059)

102 (energy adj3 (consum* or intake)).ti,ab,kw. (41514)

103 impact.ti,ab,kw. (1024510)

104 or/84-103 (2145697)

105 83 and 104 (21480)

106 limit 105 to ed=20141030-20201011 (8143)

107 exp animals/ (23721562)

108 human/ (18946304)

109 107 not 108 (4775258)

110 106 not 109 (6143)

111 letter.pt. (1116935)

112 editorial.pt. (553391)

113 comment.pt. (886056)

114 news.pt. (204715)

115 case reports.pt. (2147558)

116 111 or 112 or 113 or 114 or 115 (4037330)

117 110 not 116 (6040)

***************************

**Grey Literature/websites**

- Government websites of countries/regions known to have Govt-led restrictions on food & beverage marketing: UK, Quebec/Canada, Chile, Spain, South Korea, Ireland, Finland, Portugal
- UK Department of Culture, Media and Sport (<https://www.gov.uk/government/organisations/department-for-digital-culture-media-sport>)
- UK Department of Health and Social Care [Department of Health and Social Care - GOV.UK (www.gov.uk)](https://www.gov.uk/government/organisations/department-of-health-and-social-care)
- Regulatory and industry body websites: Ofcom, CAP, BACP, Sugar Nutrition UK, Advertising Standards Authority (ASA), Internet Advertising Bureau (IAB) UK <https://www.iabuk.com/> and Europe <https://iabeurope.eu/>, World Federation of Advertisers <https://wfanet.org/>
- Rudd Center for Food Research and Obesity at the University of Connecticut (UConn) <http://www.uconnruddcenter.org/>
- The British Retail Consortium (<https://www.brc.org.uk/>)
- Sugar reduction report (<https://www.gov.uk/government/publications/sugar-reduction-report-on-progress-between-2015-and-2019>)
- Food and Drink Federation (<https://www.fdf.org.uk/>)
- Food and Drink Federation Scotland (<https://www.fdfscotland.org.uk/>)
- Food and Drink Federation Wales (<https://www.fdf.org.uk/wales/>)
- UK Hospitality (<https://www.ukhospitality.org.uk/> )
- Market intelligence and marketing research companies: Kantar, Nielsen Europe Insights, Mintel, Lumina (<https://www.lumina-intelligence.com/> )
- World advertising research centre database <https://www.warc.com/Welcome>
- [www.science.gov/](http://www.science.gov/)
- <http://www.eHealthcareBot.com/>
- World Health Organization, <http://www.who.int/library/>
- European Commission (<https://ec.europa.eu/info/index_en>)
- European Union (<https://europa.eu/european-union/index_en>)
- Pan American Health Organisation (<https://www.paho.org/en>)
- Canadian Health Network (<http://www.canadian-health-network.ca/customtools/homee.html>)
- MedlinePlus (<http://www.nlm.nih.gov/medlineplus>)
- McKinsey and Company ([www.mckinsey.com](http://www.mckinsey.com))
- Deloitte (<https://www2.deloitte.com/uk/en/pages/tax/solutions/globaltradebureau.html> )
- National Guidelines Clearinghouse (<http://www.guideline.gov/index.asp>)
- NICE Evidence ([www.evidence.nhs.uk](http://www.evidence.nhs.uk))
- <http://worldwidescience.org/index.html>
- Action on Sugar (<http://www.actiononsugar.org/>)
- Obesity Policy Research Unit (<https://www.ucl.ac.uk/obesity-policy-research-unit/>)
- Center for Digital Democracy (CDD) (<https://www.democraticmedia.org/>)
- Obesity Health Alliance (OHA) (<http://obesityhealthalliance.org.uk/>)
- Bite Back 2030 (<https://www.biteback2030.com/>)
- Jamie Oliver group (<https://www.jamieolivergroup.com/>)
- CRUK (<https://www.cancerresearchuk.org/>)
- British Heart Foundation (<https://www.bhf.org.uk/>)
- Diabetes UK (<https://www.diabetes.org.uk/>)
- Sustain/Children’s Food Campaign (<https://www.sustainweb.org/childrensfoodcampaign/>)
- Health Equalities Group (<https://hegroup.org.uk/>)
- Irish Heart Foundation ([Irish Heart Home - Irish Heart](https://irishheart.ie/))
- eMarketer (<https://www.emarketer.com/>)
- The Drum (<https://www.thedrum.com/>)
- SuperAwesome (<https://www.superawesome.com/>)
- World Cancer Research Fund (<https://www.wcrf-uk.org/>)
- Sustain (<https://www.sustainweb.org/childrensfoodcampaign/>)
- Footprint (https://www.foodservicefootprint.com/)
- Kerry Insights (<https://www.kerry.com/insights>)
- IGD (<https://www.igd.com/>)
- Statista (<https://www.statista.com>)
- All party parliamentary Groups (APPGs) on:
  - Obesity <https://obesityappg.com/about>
  - Fit and Health Childhood <https://royalpa.co.uk/the-all-party-parliamentary-group-on-a-fit-and-healthy-childhood/>
  - Broadband and digital communication <https://publications.parliament.uk/pa/cm/cmallparty/201216/broadband-and-digital-communication.htm>
  - Food and health <https://publications.parliament.uk/pa/cm/cmallparty/201216/food-and-health.htm>

**Protocol Amendment**

Following feedback from the Expert Advisory Group for the project, an amendment to the original published PROSPERO protocol was made and approved in April 2021. The amendment stated that studies exclusively assessing the impact of advergames would no longer be excluded from the review. To address this change in criteria, searches were re-run with advergaming terms and EndNote files from the original searches and those for a concurrent World Health Organization (WHO) review^2^ by the research team were searched for advergaming terms. Retrieved articles were cross-checked against a recent meta-analysis on advergaming and dietary intake^3^ and screened as per the amended protocol.

**References**

1. Public Health England. Sugar reduction: from evidence into action. Accessible from: <https://www.gov.uk/government/publications/sugar-reduction-from-evidence-into-action>. 2015.

2. Boyland E, McGale L, Maden M, et al. Association of Food and Nonalcoholic Beverage Marketing With Children and Adolescents’ Eating Behaviors and Health: A Systematic Review and Meta-analysis. *JAMA Pediatrics* 2022; **176**(7): e221037-e.

3. Folkvord F, van 't Riet J. The persuasive effect of advergames promoting unhealthy foods among children: A meta-analysis. *Appetite* 2018; **129**: 245-51.
