# Supplementary material for: Food marketing, eating and health outcomes in children and adults: a systematic review and meta-analysis
Source: Br J Nutr. 2025 Mar 28;133(6):781–805. doi: 10.1017/S0007114524000102 (PMC12169957; doi:10.1017/S0007114524000102)
Supplement: Boyland et al. supplementary material 3 — Boyland et al. supplementary material [file S0007114524000102sup003.docx]

**Quantitative synthesis: Additional Methods**

A number of studies had more than one eligible effect size (e.g. ^(1)^). To include multiple effect sizes within studies we conducted multi-level model meta-analysis using the ‘rma.mv’ function. Study ID was used as a random intercept within the models. We attempted to identify small study / influential case analyses on the standard models (as ‘metafor’ doesn’t allow these to be conducted on multilevel models), and if any were identified we removed them from the multilevel models.

Moderation analyses (testing whether a third variable affects the relationship between the two primary variables) were conducted on the four Ps framework (Promotion, Product, Price, Place). For effect sizes on promotion, we also examined any differences by marketing type (television, digital, etc). Meta-regressions were conducted on mean age and body mass index (BMI), where available and appropriate. If there were less than 5 effect sizes in any given moderation group, it was not included.

P-curve analyses were conducted on the available effect sizes using the ‘dmetar’ package in R. P-curve examines the distribution of statistically significant two-tailed p-values (calculated from the Z score (z = effect size / standard error). If the distribution is right skewed with p-values around 0.025 to 0.050, this suggests selective reporting bias (where results from scientific research are deliberately not fully or accurately reported), whereas if the distribution is left skewed with p values < 0.025 this is suggestive of evidential value i.e., that these are true effects ^(2)^. We provide p-curve plots with statistical and visual descriptions.

For consumption, we computed the standardised mean difference [SMD = mean^exposure^ – mean^control^ / pooled SD] and the standard error. If the design was within subjects the standard error formula was adjusted by the correlation in line with Cochrane recommendations. As the correlation between measurements was not readily available we used r = 0.70, in line with previous research ^(3)^. Where a standard error was reported in place of a standard deviation, we converted it using the formula SD = SE * √N. Two studies provided effect sizes in the form of Odds Ratios, and as such we converted these to SMDs.

Given that food choice outcomes tended to be binary, we computed log odds ratios using the ‘escalc’ function in the metafor package. These log odds ratios were analysed using the same random effects models as described above. The log odds ratios were converted to odds ratios when presenting the results. The odds ratio is the odds increase of choosing the test item (advertised product or other unhealthy item) compared to alternatives.

**Quantitative synthesis: Full results**

***Consumption***

*Primary analysis*

There were 24 effect sizes extracted from 13 unique studies which measured the association between food marketing and food consumption. The multilevel meta-analysis demonstrated a significant, small effect of advertisements on subsequent consumption (SMD = 0.311 [95% CI: 0.185 to 0.437], z = 4.83, p < 0.001, I^2^ = 53.0%). A single level model demonstrated a negligibly smaller effect size (SMD = 0.296 [95% CI: 0.181 to 0.411]. There were no influential cases identified in the single level model.

*Moderation by the four Ps*

There were five effect sizes with product and 19 with promotion. There was no significant moderation effect (X^2^(1) = 0.019, p = 0.889), meaning promotion and product-based marketing produced similar effects on the outcomes. For product, the SMD was .445 [95% CI – 0.306 to 1.197] and for promotion, the SMD was .314 [95% CI: 0.182 to 0.447].

*Moderation by marketing format*

Within the promotion category there were 11 effects from TV advertising and six from digital marketing. Three effect sizes came from multiple formats and were removed from this subgroup analysis. There was no significant moderation effect (X^2^(1) = 1.683, p = 0.195) meaning that effects of TV and digital marketing on consumption were similar. For digital format the effect size was SMD = .429 [95% CI: .196 to .663], for tv format the effect size was SMD = .259 [95% CI: .067 to .449].

*Moderation by age*

Nineteen of the available effect sizes were from primarily child samples and five were from adults. There was no moderation by age (X^2^(1) = 0.827, p = 0.363) meaning effects of food marketing on consumption were similar for both age groups. For children the SMD was .338 [95% CI: 0.197 to 0.479], and for adults the SMD was 0.170 [95% CI: = -0.035 to 0.375].

Meta-regression was also used to examine whether the mean age of the sample was associated with the effect size. However, this was non-significant (coefficient = -.005 [95% CI: -.027 to .017], z = -0.444, p = 0.657).

*Moderation by study quality*

Within RCTs, five effect sizes were from ‘low risk’ studies and eight effect sizes were from studies with ‘some concerns’. There was no moderation by study quality (X^2^(1) = 0.326, p = 0.568. The effect size for ‘low risk’ studies was SMD = 0.401 [95% CI: 0.023 to 0.779], whereas the effect size for studies with ‘some concerns’ was SMD = 0.290 [95% CI: 0.037 to 0.543]

For NRS, there were 11 effect sizes with an associated quality score (ranging from 5 to 8). A meta-regression of these scores on the SMD was not significant (coefficient = -.013 [95% CI: -.141 to .155]).

*P-curve analysis*

There was limited evidence of selective reporting based on the available data. P-curve effects demonstrated evidential value (Right Skewness test, p < 0.001). The observed p-curve (**Figure S1**) includes eight statistically significant (p<.05) results, of which all eight are p<.025, indicating robust evidence of a true effect of food marketing on food consumption.

**
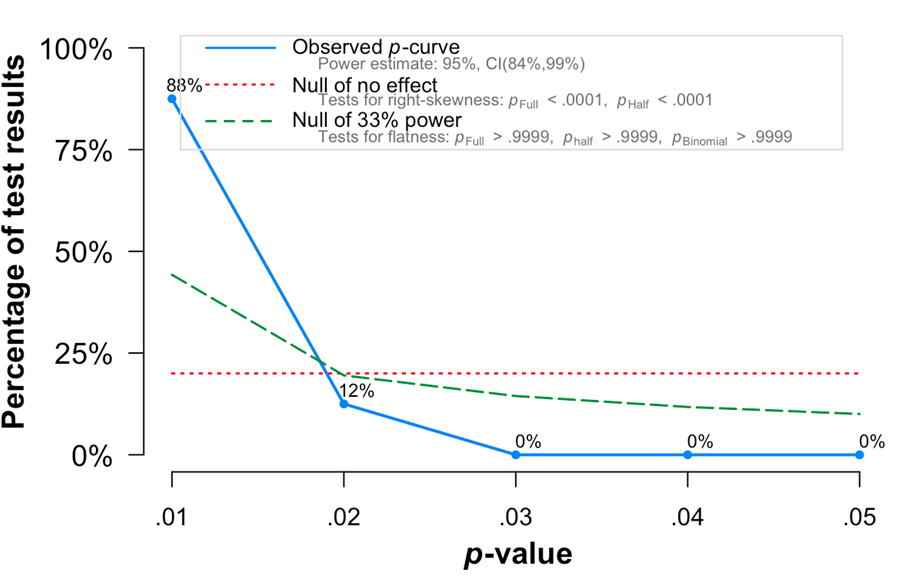
**

**Figure S1: Distribution of significant p-values from the p-curve test of the effect of food marketing on food consumption.**

***Choice***

*Primary analysis*

There were 14 effect sizes from 10 unique studies. The multilevel meta-analysis demonstrated a significant effect of advertisements on subsequent food choice (OR = 2.45 [95% CI: 1.41 to 4.27], Z = 3.18, p = 0.002, I^2^ = 93.1%). The effect size from a single level model was similar in magnitude. There were no influential cases in the single level model.

*Moderation by the four Ps*

Moderation by the four P’s was not possible as all but one effect size was for promotion. Removal of the one effect size for product did not substantially influence the overall effect (OR = 2.55 [95% CI: 1.40 to 4.66].

*Moderation by marketing format*

Within the ‘Promotion’ category there were six effect sizes for TV advertisements. The pooled effect of these studies did not show evidence of marketing impact (OR = 2.03 [95% CI: 0.78 to 5.27], Z = 1.46, p = 0.145), likely due to a lack of power.

*Moderation by age*

Moderation by age category was not possible as there was only a small number of adult studies (N = 3). As a result, only child studies were examined. The overall effect was significant (OR = 2.48 [95% CI: 1.29 to 4.82], z = 2.69, p = 0.007).

*Moderation by study quality*

Eleven of the 14 effect sizes were from RCTs, within these effect sizes all studies were rated as ‘some concerns’ except one ^(4)^ which was at low risk of bias. The remaining 10 effect sizes had an overall effect of OR = 2.02 [95% CI: 1.10 to 3.71], Z = 2.27, p = 0.023).

*P-Curve*

There was limited evidence of selective reporting based on the available data. P-curve effects demonstrated evidential value (Right Skewness test, p <0.001). The observed p-curve (**Figure S2**) includes 11 statistically significant (p<.05) results, of which 9 are p<0.025, indicating robust evidence of a true effect of food marketing on food choice.


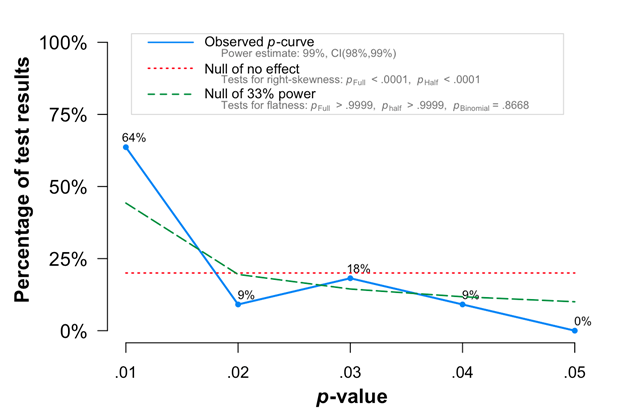


**Figure S2: Distribution of significant p-values from the p-curve test of the effect of food marketing on food choice.**

**References**

*Data and analysis scripts are available here: https://osf.io/gnck4/*

1. Aerts G, Smits T (2019) Do depicted suggestions of portion size on-pack impact how much (un)healthy food children consume. *International Journal of Consumer Studies* **43**, 237-244.

2. Simonsohn U, Nelson LD, Simmons JP (2013) P-curve: A key to the file drawer. *Journal of Experimental Psychology Available from:* [*https://papersssrncom/sol3/paperscfm?abstract_id=2256237*](https://papersssrncom/sol3/paperscfm?abstract_id=2256237).

3. Balk EM, Earley A, Patel K *et al.* (2012) Empirical Assessment of Within-Arm Correlation Imputation in Trials of Continuous Outcomes. Methods Research Report. (Prepared by the Tufts Evidence-based Practice Center under Contract No. 290-2007-10055-I.) AHRQ Publication No. 12(13)-EHC141-EF. Rockville, MD: Agency for Healthcare Research and Quality.

4. Tarabashkina L, Quester P, Crouch R (2016) Food advertising, children's food choices and obesity: interplay of cognitive defences and product evaluation: an experimental study. *International journal of obesity (2005)* **40**, 581-586.
