# Supplementary material for: Food marketing, eating and health outcomes in children and adults: a systematic review and meta-analysis
Source: Br J Nutr. 2025 Mar 28;133(6):781–805. doi: 10.1017/S0007114524000102 (PMC12169957; doi:10.1017/S0007114524000102)
Supplement: Boyland et al. supplementary material 4 — Boyland et al. supplementary material [file S0007114524000102sup004.docx]

***Modelling and simulation studies – potential impact of restrictive policies***

Four publications explored the impact of food marketing by simulating the potential effect of marketing restrictions, all four demonstrate an impact on outcomes of interest for this review.

Brown ^(1)^ determined that an intervention restricting HFSS TV advertising before 9.30pm would reduce children’s energy intake by an average of 115kJ/day (approximately 27.5kcal) and BMI by an average of 0.352kg/m^2^. These benefits would be greater in the most disadvantaged children (-132kJ/day and -0.395kg/m^2^) than in the least disadvantaged (-97kJ/day and -0.299kg/m^2^) based on differences in TV viewing time. Mytton ^(2)^ estimated that if all HFSS advertising before 9pm was withdrawn, this would decrease caloric intake by 9.1kcal (95% UI 0.5-17.7kcal) which would reduce the number of UK children (5-17 years) with overweight (including obesity) by 3.6% (95% UI 1.1%-7.4%). As with the Australian model, the UK simulation anticipated that such a policy would be more effective in the most disadvantaged groups based on differences in BMI and television viewing, with the estimated reduction in obesity approximately 2-fold greater among children in the least affluent social grade compared with the most affluent.

Dubois ^(3)^ estimated the impact of a total ban on advertising for crisps in the UK and the model predicted a 15.1% reduction in expenditure to £85.62m (95% CI 82.44-88.26) and a 15.24% fall in quantity sold to 12.55m Kg (95% CI 12.05-12.97). These reductions in purchasing were anticipated to have an impact on health, with the ban estimated to lead to a 15.23% reduction in the total quantity of energy purchased by households, from 313.70bn kJ (95% CI 310.22-316.37) to 265.94bn kJ (95% CI 256.46-274.18). Lopez ^(4)^ reported on a simulation whereby all advertising for carbonated soft drinks was prohibited found that this would lead to a decline in the market share of all such drink brands (e.g., Coke regular from 2.36% to 1.81%) and a concurrent increase in market share for alternatives (e.g., fruit juice, bottled water, milk) from 86.72% to 89.54%.

**References**

1. Brown V, Ananthapavan J, Veerman L *et al.* (2018) The Potential Cost-Effectiveness and Equity Impacts of Restricting Television Advertising of Unhealthy Food and Beverages to Australian Children. *Nutrients* **10**.

2. Mytton OT, Boyland E, Adams J *et al.* (2020) The potential health impact of restricting less-healthy food and beverage advertising on UK television between 05.30 and 21.00 hours: A modelling study. *PLOS Medicine* **17**, e1003212.

3. Dubois P, Griffith R, O'Connell M (2018) The Effects of Banning Advertising in Junk Food Markets. *Review of Economic Studies* **85**, 396-436.

4. Lopez RA, Liu YZ, Zhu C (2015) TV advertising spillovers and demand for private labels: the case of carbonated soft drinks. *Applied Economics* **47**, 2563-2576.
