# Supplementary material for: Food marketing, eating and health outcomes in children and adults: a systematic review and meta-analysis
Source: Br J Nutr. 2025 Mar 28;133(6):781–805. doi: 10.1017/S0007114524000102 (PMC12169957; doi:10.1017/S0007114524000102)
Supplement: Boyland et al. supplementary material 5 — Boyland et al. supplementary material [file S0007114524000102sup005.docx]

**Table S1: Risk of bias summary: review authors’ judgements about each risk of bias domain for included RCTs.**


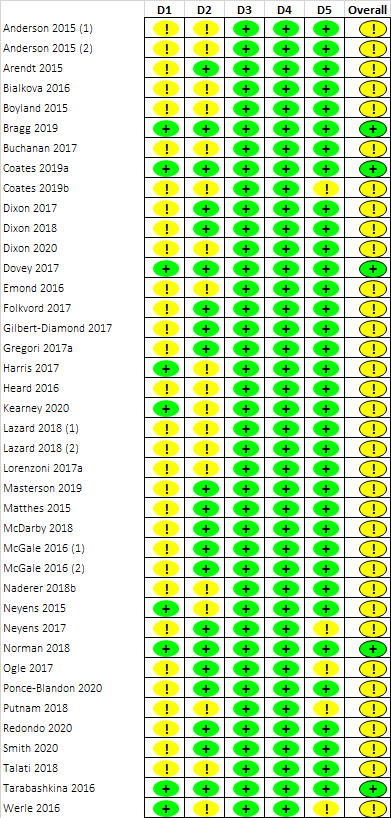


**Key:**

*Domains*

D1 Randomisation process

D2 Deviations from the intended interventions

D3 Missing outcome data

D4 Measurement of the outcome

D5 Selection of the reported result

*Risk of bias*

Low risk

Some concerns

High risk

**
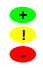
**

**Table S2: Quality Assessment summary: review authors’ judgements about each quality domain for included cross-sectional studies (NOS, maximum score 10).**

|  |  | **Selection** | | | | **Comparability** | **Outcome** | |  |
| --- | --- | --- | --- | --- | --- | --- | --- | --- | --- |
|  |  | **Representativeness of the sample** | **Sample size** | **Non-respondents** | **Ascertainment of the exposure (risk factor)** | **Comparability** | **Assessment of outcome** | **Statistical test** | **Total score** |
| Aerts (1) | 2018 | b | b | c | a | a | b | a | 8 |
| Aerts (2) | 2018 | d | b | c | a | b | b | a | 5 |
| Agante | 2019 | c | b | c | c | b | b | a | 3 |
| Baldwin | 2018 | b | b | c | c | a | b | a | 6 |
| Bollard | 2016 | b | a | c | a | b | c | a | 6 |
| Boyland | 2017 | c | b | c | a | b | b | a | 5 |
| Boyland | 2018 | b | b | c | c | a | c | a | 5 |
| Brown | 2017 | c | b | c | a | a | c | a | 6 |
| Bruce | 2016 | d | b | c | a | b | a | a | 5 |
| Buchanan | 2018 | c | b | c | c | a | c | a | 5 |
| Cohen | 2015 | b | b | b | c | b | c | a | 4 |
| Critchlow | 2020 | b | b | c | c | a | c | a | 5 |
| Dalton | 2017 | b | b | b | b | a | b | a | 7 |
| Domoff | 2021 | d | b | c | c | a | b | a | 5 |
| Egbert | 2020 | c | b | b | a | a | a | a | 7 |
| Ejlerskov | 2018 | a | b | a | a | a | b | a | 9 |
| Emond | 2019b | b | b | b | b | a | b | a | 7 |
| Forde | 2019 | b | a | a | c | a | b | a | 8 |
| Gatou | 2016 | b | b | c | a | a | b | a | 8 |
| Giese | 2015 | c | b | c | c | a | b | a | 5 |
| Girju | 2019 | b | b | c | c | a | b | a | 6 |
| Guan | 2018 | c | b | c | c | b | b | a | 3 |
| Ham | 2016 | c | b | c | a | b | c | a | 4 |
| Hennessy | 2015 | a | b | b | c | a | c | a | 5 |
| Heredia | 2017 | c | b | c | c | b | c | a | 2 |
| Kelly | 2016 | b | b | c | c | a | c | a | 5 |
| Leonard (1) | 2019 | b | b | c | b | b | b | a | 5 |
| Leonard (2) | 2019 | b | b | c | b | b | b | a | 5 |
| Leonard (3) | 2019 | b | b | c | b | b | b | a | 5 |
| Mann | 2018 | c | b | c | a | b | c | a | 4 |
| Mathe-Soulek | 2016 | b | b | c | a | a | c | a | 7 |
| Naderer | 2018a | d | b | c | a | a | b | a | 7 |
| Newman | 2020 | b | b | c | c | a | c | a | 5 |
| Nguyen | 2017 | b | b | c | a | a | c | a | 7 |
| Reimann | 2017 | b | b | c | a | b | c | a | 5 |
| Royne | 2017 | c | b | c | a | b | c | a | 4 |
| Schumacher | 2020 | d | b | c | a | a | a | a | 7 |
| Velazquez | 2016 | c | b | c | c | a | c | a | 4 |
| Yau | 2021 | b | a | c | c | a | c | a | 6 |

**Key:**

Studies shaded in grey used commercial data rather than human participant samples, so this necessitated pragmatic judgements for domains related to sampling (e.g., representativeness, size) based on information provided in the publications.

a, b, c, d = these letters denote the study quality rating for each item across all domains on the NOS scale, with ‘a’ indicating the highest quality.

**Table S3: Quality Assessment summary: review authors’ judgements about each quality domain for included cohort studies (NOS, maximum score 9).**

|  |  | **Selection** | | | | **Comparability** | | **Outcome** | | |  |
| --- | --- | --- | --- | --- | --- | --- | --- | --- | --- | --- | --- |
|  |  | **Representativeness of the exposed cohort** | **Selection of the non-exposed cohort** | **Ascertainment of exposure** | **Demonstration that outcome of interest was not present at start of study** | **Comparability a** | **Comparability b** | **Assessment of outcome** | **Was follow-up long enough for outcomes to occur?** | **Adequacy of follow up** | **Total score** |
| Emond | 2019a | b | a | c | b | a |  | c | a | b | 6 |
| Powell | 2017 | b | a | a | b | a | b | a | a | c | 7 |
| Smit | 2020 | c | a | c | b | a |  | c | a | d | 3 |

**Key:**

a, b, c, d = these letters denote the study quality rating for each item across all domains on the NOS scale, with ‘a’ indicating the highest quality.
